# Comprehenders' Error Correction Mechanisms Are Finely Calibrated to Language Production Statistics

**DOI:** 10.1111/cogs.70277

**Published:** 2026-10-30

**Authors:** Peng Qian, Roger Levy

**Affiliations:** ^1^ Department of Brain and Cognitive Sciences Massachusetts Institute of Technology

**Keywords:** Psycholinguistics, Language comprehension, Noisy‐channel language processing, Bayesian models of cognition, Rational adaptation

## Abstract

An essential feature of human communication is robustness to errors: even when speakers and writers make mistakes, listeners and readers can usually determine the intended meaning. A leading hypothesis is that comprehenders achieve robust error correction by internally deploying a “noisy‐channel” model of the language production process. Crucially, to best achieve error robustness, a comprehender's noisy‐channel model should be finely calibrated to the environmental statistics of language production, incorporating not only prior expectations about communicative intents but also structure‐sensitive likelihoods of different types of speaker error. This environmentally calibrated noisy‐channel hypothesis has been difficult to test, because the rates of most types of language production errors are hard to estimate. Here, we test this hypothesis by experimentally investigating comprehenders' interpretations of subject–verb agreement errors, for which context‐contingent language production error rates are feasible to estimate. In a novel free‐form error correction task, participants edited sentences with subject–verb agreement mismatches to indicate what they think was intended. Bayesian analysis of the resulting data shows that comprehenders' interpretations reflect both item‐specific prior expectations and structure‐sensitive error likelihoods that closely correspond to error rates in language production. These results provide quantitative evidence that humans closely track prior statistics over linguistic forms and deploy a noisy‐channel model finely calibrated to statistics of the linguistic environment to achieve robust language understanding.

## 1 Introduction

The ease with which we understand what we hear and read every day in our native language belies the challenges posed in language comprehension. Even perfectly well‐formed utterances contain ambiguous words and phrases, take time and effort to understand, and may only indirectly reflect the speaker or writer's communicative intent. Wide‐ranging evidence suggests that comprehenders use probabilistic inference to surmount the challenges posed by well‐formed utterances (Bod, Hay, & Jannedy, 2003; Bresnan, Featherston, & Sternefeld, 2007; Chater & Manning, 2006; Jurafsky, 1996). In ambiguity resolution, human interpretive preferences reflect the structural statistics of linguistic experience (Brysbaert & Mitchell, 1996; Cuetos, Mitchell, & Corley, 1996; D. C. Mitchell & Cuetos, 1991). Prediction of upcoming linguistic input is ubiquitous and facilitates rapid, efficient comprehension (Federmeier, Wlotko, De Ochoa‐Dewald, & Kutas, 2007; Kutas & Federmeier, 2011; Kuperberg & Jaeger, 2016; Levy, 2008a; N. J. Smith & Levy, 2013); models of linguistic prediction also account well for brain responses during language comprehension (Gauthier & Levy, 2019; Goldstein et al., 2022; Heilbron, Armeni, Schoffelen, Hagoort, & De Lange, 2022; Schrimpf et al., 2021). Pragmatically sophisticated language understanding has also been modeled as probabilistic inference among cooperative agents (Franke & Bergen, 2020; Goodman & Frank, 2016). These findings support the general thesis that language comprehension is rationally organized (Anderson, 1991) and well adapted to the patterns of everyday language use—that is, the linguistic environment.

Of course, not all utterances are perfectly well‐formed. Speakers and writers are imperfect, and errors are pervasive. For example, people may mispronounce words, utter fragmentary sentences, make typographical errors, etc. Yet, these errors rarely impede successful understanding. Previous work has theorized that comprehenders achieve robust language understanding by internally deploying a Bayesian “noisy‐channel” model of how language production errors distort a speaker or writer's communicative intent to yield the comprehender's observed utterance (Gibson, Bergen, & Piantadosi, 2013; Levy, 2008b; Shannon, 1948):

$$\begin{array}{c} \overset{\text{Posterior}}{\overset{︷}{P(\text{intent}|\text{observation})}}\propto \overset{\text{Likelihood}}{\overset{︷}{P(\text{observation}|\text{intent})}}\overset{\text{Prior}}{\overset{︷}{P(\text{intent})}} \end{array}$$

with the prior probability taking into account a comprehender's broader knowledge and contextual expectations about the speaker's likely communicative intent, and the likelihood being higher the fewer and more common the errors that would need to be hypothesized to reconcile the intent with the observed utterance. According to the theory, comprehenders will favor interpretations that jointly maximize this posterior probability, balancing prior and likelihood. If comprehension involves noisy‐channel processing with probabilities that are calibrated—that is, quantitatively well‐matched—to fine‐grained statistics of the linguistic environment, including structure‐sensitive probabilities of different types of language production errors, then it would offer an explanation of how robust language understanding is achieved, identifying and correcting the numerous errors encountered in everyday language in an optimal manner. This notion of optimality relates to the idea of a Bayes optimal classifier in the machine learning literature (T. M. Mitchell et al., 1997). We call this the *environmentally calibrated noisy‐channel theory*.

A number of studies have tested and confirmed qualitative predictions of the noisy‐channel theory: effects of structural frequency and utterance plausibility (corresponding to prior probability), a preference for positing minimal numbers of errors, and sensitivity to the overall rate of implausibility and errors in an experiment (Brehm, Jackson, & Miller, 2019; Gibson et al., 2013; Gibson et al., 2017; Keshev & Meltzer‐Asscher, 2021; Poppels & Levy, 2016; Ryskin, Futrell, Kiran, & Gibson, 2018). But a strong test of the environmentally calibrated noisy‐channel theory would involve quantitative comparisons of the parameters of the comprehender's internal model with the relevant statistics of the linguistic environment, in particular the error rates in language production. This is a challenging problem, because while language production errors are collectively common, most *types* of errors are individually rare, making it difficult to estimate their frequencies. Furthermore, many types of errors can be corrected in widely varying ways, making it difficult to estimate the probabilities in a comprehender's internal noisy‐channel model.

In the present work, we surmount these challenges by focusing on a type of language production error that is particularly amenable to quantitative analysis within the noisy‐channel approach: subject–verb agreement errors, such as in sentence [1] below:

[1] The gift for the kids are hidden under the bed.

where the head noun of the subject, *gift*, is singular but the verb, *are*, is plural. Since the inception of contemporary linguistic theory, subject–verb agreement has played a central role (Chomsky, 1957). When the subject is structurally complex, agreement errors can be frequent enough to study quantitatively; for example, when the noun adjacent to the verb (the “local” noun) differs in number from the head noun of the subject, as in example [1], agreement errors are more likely (Bock & Miller, 1991). The factors promoting or inhibiting agreement error rates have been a topic of considerable study in language production research (Barker, Nicol, & Garrett, 2001; Brehm & Bock, 2013, 2017; Brehm, Cho, Smolensky, & Goldrick, 2022; Eberhard, 1997; Eberhard, Cutting, & Bock, 2005; Franck, Vigliocco, & Nicol, 2002; Franck, Vigliocco, Antón‐Méndez, Collina, & Frauenfelder, 2008; Gillespie & Pearlmutter, 2011; Haskell, Thornton, & MacDonald, 2010; Humphreys & Bock, 2005; Hartsuiker & Barkhuysen, 2006; Kandel et al., 2022; Thornton & MacDonald, 2003; Vigliocco, Butterworth, & Semenza, 1995; Veenstra, Antoniou, Katsos, & Kissine, 2018, inter alia). Additionally, as the experimental data we present here will show (see also Brehm et al., 2019, 2021; Patson & Husband, 2016), when comprehenders are asked to correct agreement errors, the space of likely corrections is highly constrained, making it feasible to estimate comprehender model parameters. Here, we provide a computational implementation of the noisy‐channel theory, estimate its parameters from experimental data, and compare those parameters to estimated error rates from experimental language production data to adjudicate whether noisy‐channel comprehension is finely calibrated to the statistics of the linguistic environment.

To illustrate the factors at play in comprehension of subject–verb agreement errors, note that, for example [1], there are multiple possible inferences about what the speaker intended. The speaker might have produced the subject phrase *the gift for the kids* correctly but mistakenly inflected the verb as *are* instead of an intended *is*; alternatively, the speaker might have intended a plural head subject noun, *gifts*, but misinflected it as *gift*. Noisy‐channel theory predicts that error correction preferences should depend quantitatively on the prior probabilities of the specific candidate error‐free forms. In example [1], this prior would reflect how likely one would be to communicate a situation where there are multiple gifts for multiple kids, versus just one gift for multiple kids. According to our experimental estimates, the former situation is considered somewhat more likely than the latter. In addition to this effect of the prior, the theory predicts that error correction preferences should also depend on the likelihood of different kinds of errors. In the example, the competing likelihoods are of misinflecting an intended plural noun as singular (*gifts* → *gift*), versus misinflecting an intended singular verb as plural (*is* → *are*). According to our analysis of previous results from the language production literature, the latter error is more likely than the former (Kandel et al., 2022; Ryskin, Bergen, & Gibson, 2021). In example [1], prior and likelihood thus bias the comprehender toward different interpretations, and each interpretation about the speaker's intent will be reasonably likely. In contrast, in example [2] below:

[2] The rug under the chairs are handmade from wool and silk.

the prior strongly favors a situation involving a single rug. For example [2], the prior and likelihood thus both strongly favor the inference that the speaker's error was in producing the verb as *are* instead of *is*, so we should see an accordingly strong preference for this interpretation.

The logic described above follows from a fully rational Bayesian noisy‐channel model as illustrated in the bottom right of Fig. 4, which specifies a joint distribution P(E,M,O) over communicative intents M generated according to the prior P(M), errors E generated given the specific intended message M, and a resulting observed sentence O generated by applying any error(s) E to M. Comprehenders face the problem of reverse inference: given an observed sentence with agreement mismatch O, to compute P(M,E|O): the posterior distribution over the speaker's intended message M and language production errors E. However, more limited forms of noisy‐channel Bayesian inference are also possible, as illustrated in Fig. 4: the comprehender might take into account only one of the prior or likelihood, or might take into account both but use error likelihoods that are insensitive to structural features of the grammatical context. Nevertheless, all of these alternative models would be suboptimal for accurately inferring speaker intent, because they would fail to take into account features of the linguistic environment that have predictive value: intended message probabilities are non‐uniform, some language production errors are more likely than others, and the grammatical context affects error rates.

**Fig. 1:**
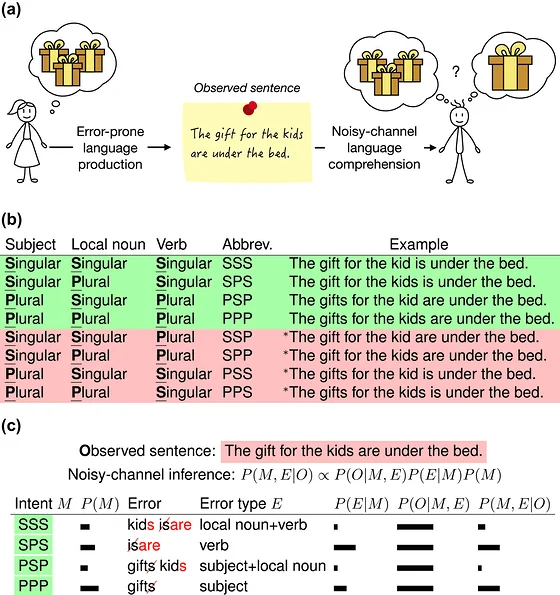
(A) A sketch of error‐prone language production and noisy‐channel error correction in everyday communication. (B) The eight logically possible combinations of {Singular,Plural} number features for the head noun of the subject, the noun linearly local (adjacent) to the main verb, and the verb. Four of these combinations adhere to the grammatical rule of English requiring that the subject and verb agree in number; the other four conditions, marked with 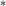, violate this rule. The four rule‐following combinations constitute possible communicative intents M that comprehenders may infer when encountering a rule‐violating sentence. The four rule‐violating combinations constitute the four conditions of our experiments, which are observations O in the noisy‐channel theory. (C) The logic of Bayesian inference within the noisy‐channel theory, illustrated for O=“The gift for the children are under the bed” (SPP condition), which could have arisen due to different language production errors for any of the four possible communicative intents M. Comprehenders integrate prior expectations about communicative intent, P(M), with context‐contingent likelihoods of the language production errors E that would be required to generate the observed sentence O from M. Given E and M, O is deterministic, hence P(O|E,M)=1. A comprehender's posterior belief regarding communicative intent and language production errors is given by Bayes' rule: P(M,E|O)∝P(O|E,M)P(E|M)P(M).

**Fig. 2:**
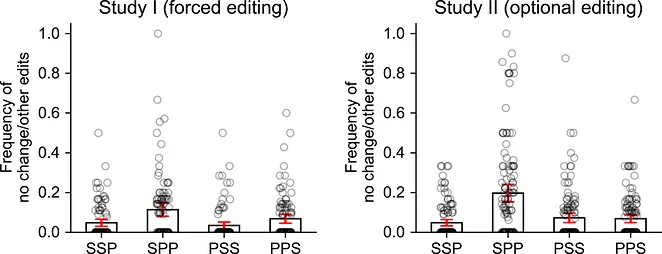
Proportion of trials in the error correction tasks (Study I and II) that did not resolve agreement mismatch. The labels on the horizontal axis show four trial conditions. Hollow circles represent participant‐specific responses.

**Fig. 3:**
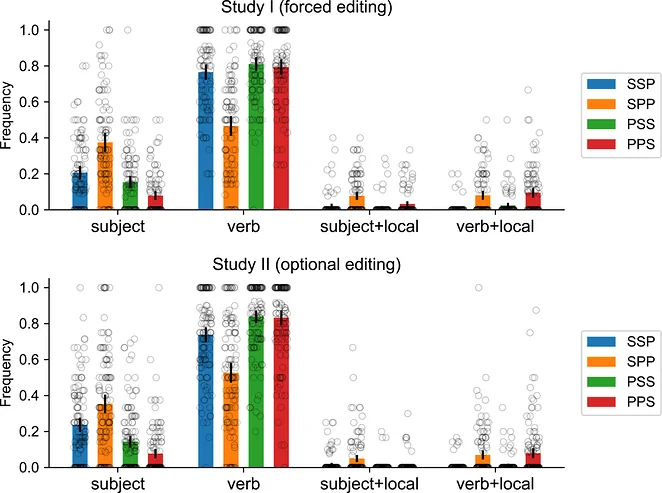
Distribution of the type of error corrections in Study I and Study II for trials that resolved subject–verb number mismatches. Hollow circles represent participant‐specific responses.

**Fig. 4:**
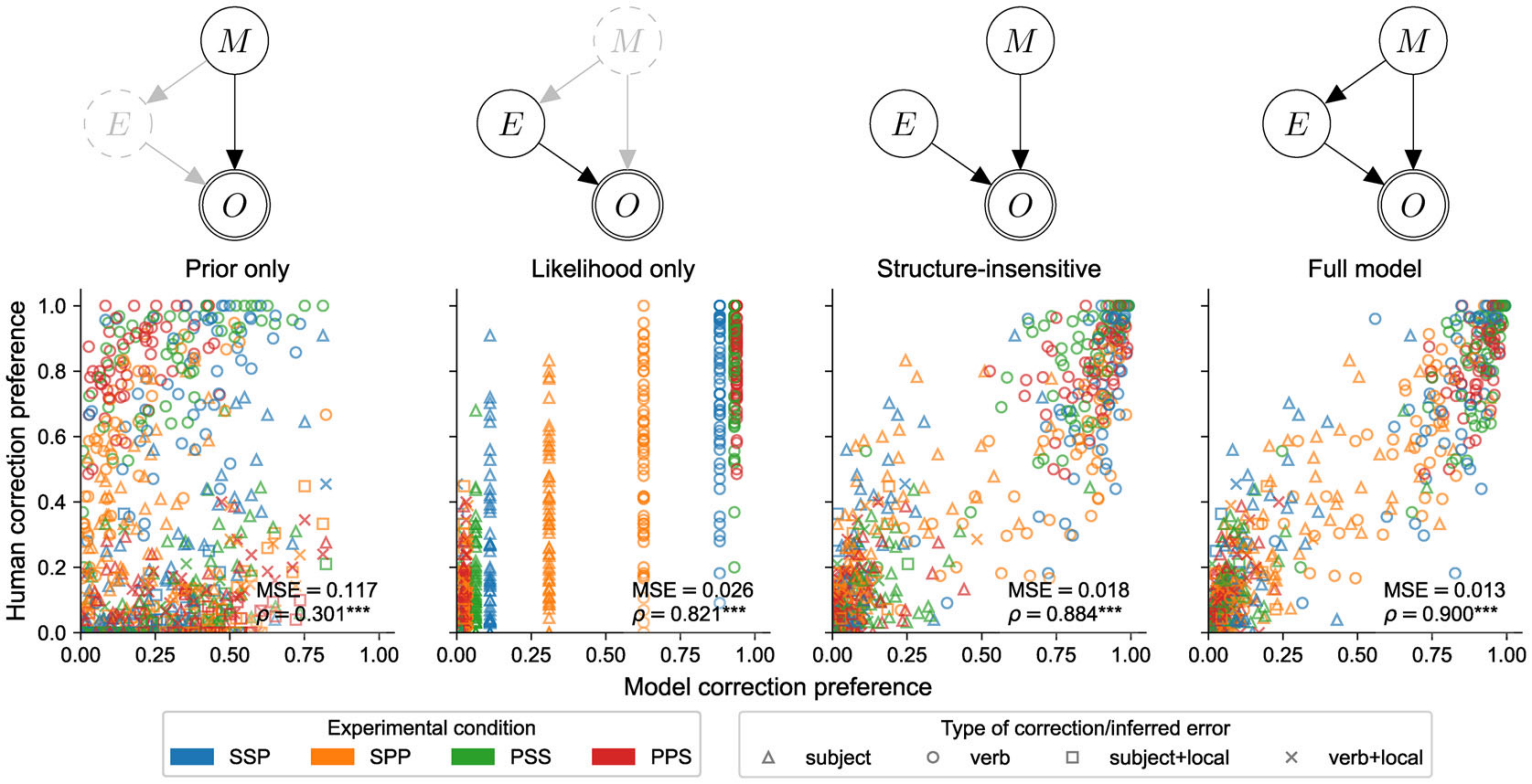
Top: Candidate generative models underlying inference in robust language understanding. Here, M refers to the intended message, E refers to the type of production error, and O refers to the utterance observed by the comprehender. The double circle around O indicates that the conditional distribution given its parents M and E is deterministic: given an intended utterance M and production error(s) E, the actual utterance is determined by applying E to M. Dashed‐circle nodes are modeled with a uniform distribution; solid single‐circle nodes are modeled as context‐sensitive according to the statistics of the linguistic environment. Bottom: Comparison of how well each of the four fitted candidate models' predictions matches comprehenders' error‐correction preferences in our experiments. For an explanation of experimental conditions and inferred error types, see Fig. 1.

Our experiments further contrast the environmentally calibrated noisy‐channel hypothesis with previous noisy‐channel theorizing regarding the comprehender's likelihood model, in which simplicity—a preference for inferring fewer and smaller errors—plays the dominant role (Gibson et al., 2013; Levy, 2008b). Consider in the above examples the relative likelihoods of misinflecting a morphophonologically regular subject noun (e.g., *gifts* → *gift* in Example [1]) versus misinflecting the verb *is* as *are*. From orthographic and phonological perspectives, misinflecting the verb is a “larger” error than misinflecting the noun, which is only a single‐letter or single‐phoneme change. From the perspective of word‐level substitutions, the two types of misinflections would be equivalent. Thus, simplicity‐based principles (Chater & Vitányi, 2003; Feldman, 2003) would predict that comprehenders assign at least as high a noisy‐channel likelihood to regular noun misinflection as to verb misinflection. In actual language production statistics, however, verb misinflections are more likely.

The environmentally calibrated noisy‐channel theory thus makes four key predictions for interpretations of errorful sentences that we test here. First, interpretation preferences should reflect prior expectations about the message the speaker may have intended. Second, interpretation preferences should also reflect the likelihoods of the different kinds of errors that need to be posited in order to explain the errorful sentence. Third, these error likelihoods should be sensitive to structural features of the intended utterance's structure. Fourth, these error likelihoods should be well calibrated to actual error rates in language production—in contrast with the simplicity‐based noisy‐channel hypothesis, which predicts a discrepancy between the two regarding the relative probabilities of subject–noun inflection errors and verb inflection errors.

We test these predictions by analyzing and modeling human behaviors in correcting English sentences with subject–verb number agreement mismatches. We elicit free‐form corrections from human participants in two error‐correction tasks. We further estimate prior expectations through an independent norming study. The frequency distribution over correction types and prior judgment of related variants of the observed stimulus indicates a strong influence of prior on error correction behaviors. Statistical analysis confirms effects of both prior and context‐sensitive error likelihoods as predicted by the full model and shows that the estimated noisy‐channel error likelihood parameters are broadly consistent with those reported in empirical studies of language production errors. These results support the environmentally calibrated noisy‐channel hypothesis and suggest that comprehenders closely track prior statistics and fine‐grained, structure‐conditioned error rates in the linguistic environment.

### 1.1 An overview of the studies and analyses

To examine the predictions of this computational‐level account of robust language comprehension, we planned a series of studies and analyses. To start with, we conducted two error correction studies (Study I and II) in order to measure people's preferences in resolving subject–verb number mismatches. In the framework of the noisy‐channel processing theory, people's responses to the error correction tasks would indicate their inferences about the communicative intent as well as the error that had occurred to the intended message during production. Following the descriptions of people's behaviors in the error correction task, we present a detailed characterization of the full computational model as well as the ablated versions for alternative hypotheses, which motivate the subsequent analyses of the prior and error likelihood. Next, we conducted a norming study to measure people's prior expectation over relevant error‐free forms. This allowed us to compare people's prior with the distribution of the types of correction, as an examination of the prior effect in noisy‐channel inferences. Last but not least, we drew statistical inferences about the error likelihood involved in the noisy‐channel error correction process, given the empirical measures of the prior and correction preferences. We conducted a reanalysis of the available language production datasets that target subject–verb number agreement errors. To answer the research question about the calibration of error models, we examined whether the error type distribution that people deployed in the error correction process tracks the differential patterns of error types in language production.

## 2 People's behaviors in correcting subject–verb number mismatches

We first measured people's preferences for various possible ways of resolving subject–verb grammatical number mismatches. To do this, we conducted two behavioral studies that elicited free‐form error correction behaviors from participants.

### 2.1 Study I: Free‐form error correction

#### 2.1.1 Methods

##### 2.1.1.1 Stimuli

We designed a set of target items and filler items. The **target items** use “The [$N_{1}$] [Preposition] the [$N_{2}$]” as the structure of the subject phrase but vary in semantic content. The complete combination of nouns $N_{1}$ and $N_{2}$ based on the choice of singular or plural features, together with a grammatically number‐mismatched verb, results in four conditions as shown in the bottom four rows of Fig. 1b. The four conditions correspond to different combinations of singular (S) and plural (P) variants of the subject noun ($N_{1}$) and local noun ($N_{2}$). We refer to these conditions as SSP, SPP, PSS, and PPS, based on the number feature of the head noun ($N_{1}$), the local intervening noun ($N_{2}$), and the verb. We also use SSS, SPS, PSP, and PPP to refer to the structure of the communicative intent if there were no errors during utterance production.

The **filler items** for the study contain various kinds of errors beyond number agreement mismatches, including errors in the use of articles, repetition of function words, and fragmentary structures, etc.

We initially designed 57 target items. Later, we noticed a problem in the presentation of one particular item in the experiment due to missing the plural suffix for the supposed plural version of the local noun in the item. Hence, only data for the remaining 56 items were included in the analyses. Some of the target items were adapted from prior works on agreement errors (Bock & Miller, 1991). All the 56 target items and 30 filler items are included in the Supporting Information.

##### 2.1.1.2 Participants

We recruited 99 native English speakers online from the United States on Prolific (www.prolific.co), a crowdsourcing platform. The sample size was determined by convenience and based on previous experience. Informed consent was obtained from every participant. One participant was recruited twice, so we excluded responses from that participant when completing the study for the second time.

##### 2.1.1.3 Experimental procedure

The goal of the free‐form error correction task was to measure human's error correction preferences. In this task, participants were asked to correct the mistake(s) in the presented sentence while respecting the intended message by the original writer.^1^ In the free‐form error correction study, participants saw 30 fillers and a random subset of 30 target items. Items were presented in random order, with one item per trial. For every participant, each target item in the error correction task was presented randomly in only one of the four mismatch conditions: SSP, SSP, PSS, and PPS, as shown in the bottom four rows of Fig. 1b. Each participant saw a roughly similar number of trials for each condition in a task. The experiment was implemented with jsPsych (De Leeuw, 2015). The study received IRB approval from the university.

Participants were required to perform at least one edit on the presented sentence in a trial before proceeding to the next trial. We collected the edited sentences for each trial in the experiment. The free‐form editing responses were then mapped to the corresponding type of correction across “subject,” “verb,” “subject+local,” and “verb+local” (see Fig. 1c), resulting in a distribution over the type of errors that participants inferred from the observed subject–verb number agreement mismatch.

##### 2.1.1.4 Coding the types of corrections

We mapped participants' free‐form error correction responses to a feature‐based representation, which is then compared to the trial condition to identify the specific *type* of correction for each trial. This feature‐based representation focuses on changes to the number feature of the nouns in the subject phrase and the verb. For example, a stimulus of condition SPP, “The gift for the kids are hidden under the bed.” might be edited into “The gifts for the kid are hidden under the bed.” of which the post‐edit version consists of a plural head noun of the subject, a singular local noun, and a plural verb. This correction will be identified as “SPP →PSP,” referred to as “subject+local” correction, indicating that the participant inferred errors with both the subject head noun ($N_{1}$) and the local noun ($N_{2}$). Table 1 lists some other examples of subject correction, verb correction, subject+local correction, and verb+local correction. We implemented the coding of error correction type through a rule‐based function with the support of the nltk part‐of‐speech tagger (Loper & Bird, 2002).^2^

**Table 1:** Examples of error correction and their corresponding type

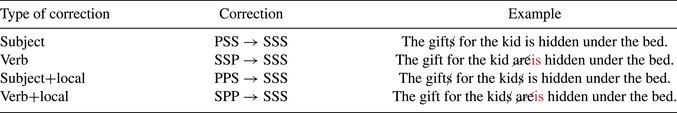	

#### 2.1.2 Results

We analyzed the distribution of the type of error correction choices among participants. For Study I, among all the trials that presented the target items, 6.501% did not resolve the agreement mismatch. In these trials, participants edited other parts of the sentence, or changed the local noun alone without addressing the agreement mismatch. We plot the frequency of such trials that did not explicitly resolve agreement mismatches in Fig. 2. We observe that participants more often left the mismatch unaddressed given the SPP condition than other conditions (p=.0226), which is consistent with previous studies that suggest the presence of a plural attractor increases the likelihood of agreement error (Bock & Miller, 1991) and an agreement mismatch with a plural attractor induces less processing difficulty in sentence comprehension than that with a singular attractor (Wagers, Lau, & Phillips, 2009).

Fig. 3 shows the frequency of editing types averaged across subjects for the trials whose agreement mismatches were corrected. Overall, humans show a strong preference for changing the verb across conditions. The rate of subject correction varies across mismatch conditions. We compare the observed pattern of the correction type distribution to relevant results reported in previous works. Patson and Husband (2016) combined self‐paced reading with a forced‐choice comprehension question paradigm to study the effect of agreement attraction on non‐literal interpretation of the number feature of the subject noun, focusing on the case of a singular subject with a plural attractor. The percentage of non‐literal interpretation of the subject number feature was about 22% for SSP, about 35–45% (estimated from two experiments) for SPP, and about 15% for PPS. Brehm et al. (2019) reported comprehension studies with an experimental paradigm similar to that of Patson and Husband (2016), focusing on the speaker‐specific effect on non‐literal interpretation. The overall pattern across conditions is similar: the proportion of interpreting a singular subject as plural was about 20% for SSP and about 40% for SPP. With a visual world paradigm and different stimulus modalities, results from Brehm, Jackson, & Miller (2021) also suggested that the presence of a plural attractor (SPP as compared to SSP) would increase the rate of non‐literal interpretation of the subject number feature. Results from our free‐form editing experiment, as shown in Fig. 3, are consistent with those of the corresponding conditions in previous research (Brehm et al., 2019, 2021; Patson and Husband, 2016) and complement prior work with additional data for the case of PSS, plural subject with singular attractor.

### 2.2 Study II: Optional free‐form error correction

The error correction task in Study I required the participants to make some edits to the input for every trial in the experiment. One concern with this task is that people's inferences from agreement mismatches may be influenced by strategic behavior on the part of the participants. To address this concern, we conducted a second study where editing is optional.

#### 2.2.1 Methods

##### 2.2.1.1 Stimuli

We used the same set of 56 target items and 30 filler items from Study I and followed the same experimental setup for randomly sampling a subset of 30 target items for each participant.

##### 2.2.1.2 Participants

We recruited 120 native English speakers online from the United States on Prolific. The sample size was determined by convenience and was chosen to be larger than Study 1, as we expected that participants may skip some trials as intended by the experimental design. Informed consent was obtained from every participant.

##### 2.2.1.3 Experimental procedure and response coding

The general procedure of this study was the same as Study I, except that participants were given an option to skip a trial by checking a box that explicitly says “no edit is needed” during the trial. Participants were shown the same instructions as for the free‐form error correction task in Study I, with additional information on how to skip a trial. The study received IRB approval from the university. For the target trials, participants' responses were mapped onto the four types of corrections (subject, verb, subject+local, verb+local) in the same way as Study I.

#### 2.2.2 Results

Compared to Study I, we observed a slightly higher rate (9.853%) of responses that explicitly skipped the trials or performed edits that do not target the agreement mismatch in Study II, as shown in Fig. 2. Taking a closer look at the distribution of responses that do not target the agreement mismatch, we see that the SPP condition has the highest rate of not being corrected (p<.001). This pattern matched the observation from Study I, suggesting that while making the edit optional leads to increased responses that do not identify the agreement mismatch, the relative sensitivity to the identification of the error remains similar. For the trials in Study II that did resolve the agreement mismatch, we performed the same set of visualizations and analysis as Study I, as shown in Fig. 3.

## 3 Computational models

Our studies and analyses are motivated by a formal model of noisy‐channel inference (Gibson et al., 2013; Levy, 2008b; Levy, Bicknell, Slattery, & Rayner, 2009). As we elaborate here, this computational proposal hypothesizes that people represent a generative model of how intended messages may be corrupted by errors and result in sentences with number agreement mismatches. Robust understanding of noisy and erroneous linguistic input then involves inverting the generative process to infer the likely error and the intended message, conditioned on the recognized number agreement mismatch in the sentence. We note that the general conceptual idea that a rational agent deploys an internal model of the speaker in language comprehension also relates to models of pragmatic reasoning such as the Rational Speech Act framework (Frank & Goodman, 2012).

We built Bayesian models for the error correction studies, assuming that a rational language user reverses the generative process to infer the cause of agreement mismatches and perform targeted correction to fix the inferred type of errors. As described earlier, we assume a probabilistic generative model P(E,M,O) over intent M, error type E, and observation of agreement mismatch O. For models shown in Fig. 4, M takes a value in SSS, SPS, PSP, PPP, E takes a value in subject, verb, subject+local, and verb+local, and O takes a value in SSP, SPP, PSS, and PPS.

For the full model, the forward generative process is as follows: an intended message M is sampled from a prior distribution P(M). Then an error E is sampled from P(E|M), conditioned on the structure of the specific message.^3^ Given M and E, O is uniquely determined, as formalized by the indicator function 1(O|M,E) (see Table S1 in the Supporting Information for an elaborated representation). For example, a possible value of M could be “The gifts for the kids are…” (PPP), which is sampled from the prior P(M) over the four possible error‐free versions: “The gift for the kid is…” (SSS), “The gift for the kids is…” (SPS), “The gifts for the kid are…” (PSP), and “The gifts for the kids are…” (PPP). For comprehenders, the problem is reverse inference: given an observation with agreement mismatch O, compute P(E|O), the posterior distribution of error type E given the observation O. With the parameterization of Θ as the error likelihood and α as the temperature parameter of the prior in the model, the posterior P(E|O) can be elaborated as

(1) $$\begin{array}{cc} P(E|O;\alpha ,\Theta ) & =\frac{P(E,O;\alpha ,\Theta )}{P(O;\alpha ,\Theta )}\propto \sum_{M}P\left(E,M,O;\alpha ,\Theta \right) \end{array}$$

(2) $$\begin{array}{cc} & =\sum_{M}1\left(O|M,E\right)P\left(E|M;\Theta \right){\left[P(M)\right]}^{\alpha }. \end{array}$$

Note that while the latent variable M is marginalized out in Eq. 2, only one specific value of M will result in a non‐zero joint probability during the summation due to the deterministic mapping between O and a particular combination of M and E as specified by the indicator function 1(O|M,E). The temperature parameter α weights the contribution of the prior, as independently estimated from the norming task data. The posterior over α will indicate how strong the effect of prior judgment over intended linguistic forms is on humans' inference of errors, serving as a test of the effect of the prior. We model humans' correction choices as being chosen probabilistically according to the posterior distribution of potential error types P(E|O;α,Θ).

In addition to the full Bayesian model with structure‐sensitive parameterization of the error likelihood, we also consider ablated models as alternative hypotheses, as introduced in Fig. 4. The “prior only” model and the “likelihood only” model consider the variation in human correction behaviors as explained solely by the variation in the prior across items or the error likelihood, respectively. We derive the correction preference from the “prior only” model as

(3) $$\begin{array}{c} P\left(E|O;\alpha \right)\propto \sum_{M}1\left(O|M,E\right){\left[P\left(M\right)\right]}^{\alpha }. \end{array}$$

We derive the correction preference from the “likelihood only” model as

(4) $$\begin{array}{c} P\left(E|O;\Theta \right)\propto \sum_{M}1\left(O|M,E\right)P\left(E|M;\Theta \right). \end{array}$$

To further test the hypothesis that the shape of the likelihood varies across specific structural contexts of the intended message, we implemented an alternative Bayesian model that parameterizes the likelihood Θ with structure‐insensitive probability of each type of error shared across different values of M, leaving three free parameters in the likelihood of the model to be estimated from data. This corresponds to the “structure‐insensitive” hypothesis in Fig. 4. For this model, the posterior over error type E is derived as follows after applying Bayes' rule:

(5) $$\begin{array}{c} P\left(E|O;\alpha ,\Theta \right)\propto \sum_{M}1\left(O|M,E\right)P\left(E;\Theta \right){\left[P\left(M\right)\right]}^{\alpha }. \end{array}$$

Note that the error likelihood P(E) of the context‐insensitive model in Eq. 5 is not conditioned on the specific value of the communicative intent M. Similar to the computation of the posterior P(E|O) of the full model illustrated in Eq. 2, we add the additional temperature parameter to the prior part in Eq. 5 as well.

For all the models, we add the same random‐effect structure to account for potential variability across items and participants, in addition to the fixed effect discussed above. Random intercepts for item–condition combinations as well as participant–condition combinations are added to log(P(E|O)) and were estimated together with the fixed effect. We implemented the models in Stan (Carpenter et al., 2017) and performed Bayesian estimation of the parameters (α, Θ) with a weakly informative prior, using the error correction data collected in Study I and II and the priors measured in a norming study (see details in Section 4.2). We ran Markov chain Monte Carlo‐based approximate inference with four chains of 10,000 iterations, with the first 2,000 iterations as warm‐up.

## 4 Evidence for calibrated noisy‐channel inferences

Here we report analyses and results based on combined data from Study I and II for brevity, since Study II was designed as a replication of Study I. Separate analyses of the data from each study show similar quantitative results and are included in the Supporting Information. Modeling results were based on data from the trials where participants resolved the number mismatch between the subject and the verb in the target items. As was previously discussed, the vast majority of human responses resolved agreement mismatches in both studies (see Fig. 2).

### 4.1 Model comparison

Fig. 4 compares the human correction preference to the correction preference derived from the models fitted on the human data (see Section 3 for details). We evaluated models based on the mean squared errors (MSE) between the model and human. The full Bayesian model achieves the lowest MSE. We observe significant correlation between human behaviors and the prior as well as the likelihood, suggesting that both prior and likelihood contribute to the explanation of human correction choices. However, neither of these two components alone offers the best account of human behavior, as indicated by the lower Spearman correlation coefficients and higher MSE in comparison to the full model. The structure‐insensitive model provides a better fit to the human behavioral data than the prior only and likelihood only models, but still not as good as the full model. We compared the full model and the structure‐insensitive model using a likelihood ratio test, a versatile hypothesis testing procedure for comparing two alternative hypotheses of which one is nested within the other. The result of the likelihood ratio test of the structure‐insensitive model with constrained likelihood parameterization and the full model rejects the null hypothesis of structure‐insensitive likelihood parameters ($\chi^{2}\left(9\right)=308.66$, $p=3.79\times 10^{-61}$).

In addition, we estimated the expected log pointwise predictive density (ELPD) for the full model and structure‐insensitive model using K‐fold cross‐validation (Vehtari, Gelman, & Gabry, 2017). We partitioned the data of each study into eight folds by repeatedly holding out complete trial data within a random set of seven items (see Section A in Supporting Information for details of the statistical procedure). We found noteworthy ELPD differences between the structure‐insensitive model and the full model across Study I (ΔELPD=−41.71, SE=10.84) and Study II (ΔELPD=−58.29, SE=12.06). Breaking down the data by specific stimulus type, we saw consistently large ELPD differences for trials of the SPP condition across Study I (ΔELPD=−25.58, SE=7.46) and Study II (ΔELPD=−18.99, SE=6.29). These results suggest that the full model provides better generalization performance than the structure‐insensitive model.

### 4.2 Effect of prior

#### 4.2.1 Norming study

We conducted a norming study to estimate the prior preference over the four possible combinations of subject noun and local noun for each target item, independent of the error correction studies. In the norming study, the items were kept the same except that the main verb was presented in a neutral form without number inflection, such as “The gift for the kid might be hidden under the bed.” in order to avoid potential attraction effect in comprehension (Pearlmutter, Garnsey, & Bock, 1999; Patson and Husband, 2016; Wagers et al., 2009). Participants in the norming study were asked to judge how likely they think someone might say each variant of an item on a slider scale of 1 to 100, where 1 indicates “extremely unlikely” and 100 indicates “extremely likely.” The four conditions of an item were presented altogether on the same page to reduce the possibility of misperceiving the number feature, in random order across participants. Participants moved the slider bar for each of the four conditions of an item during a trial. For a complete documentation, participants in the norming task also responded to additional multiple‐choice trials in a separate block, where they were asked to choose the most likely combination of the singular/plural variant for the subject and local noun given the same set of items used in this study, such as “The [gift/gifts] for the [kid/kids] might be hidden under the bed.” Responses to these multiple‐choice trials were not part of the analyses here. We note that the frequency distribution of participants' choices was consistent and highly correlated with the averaged rating of likelihood from the norming trials.

We recruited 20 native English speakers on Prolific for the norming study. Data from 6 participants were excluded due to failure to meet the rating criterion of an attention check trial. The study received IRB approval from the university. Each participant's rating data for the four error‐free versions (SSS, SPS, PSP, PPP) of an item were normalized into a probability distribution. The mean normalized rating averaged across participants is used as the estimated prior over four conditions of the intended messages for the corresponding item.

##### 4.2.1.1 Sensitivity to fine‐grained patterns in prior expectations

We now investigate how prior plausibility over the intended content relates to error correction preferences. One simple way to do this is to examine the relationship between the prior and how often participants correct the number of the verb. Intuitively, the greater the prior probability of the observed form of the subject phrase, then the less likely there is to have been an error in its production, implying that it must have been the verb that was misinflected. Fig. 5 shows the verb correction rate against the prior of the error‐free sentence with the same subject phrase as the observation, which is estimated from the norming study. For example, the verb correction frequency in human responses for an input of the mismatch condition SPP, such as “The gift for the kids are hidden under the bed.”, is plotted against the estimated prior of the corresponding error‐free version, such as “The gift for the kids will be hidden under the bed.” Fig. 5 plots data from all four mismatch conditions together. We observe significant correlation between the verb correction rate and the prior of the error‐free sentence with the same subject phrase (Spearman ρ=0.602, p<.001).

**Fig. 5:**
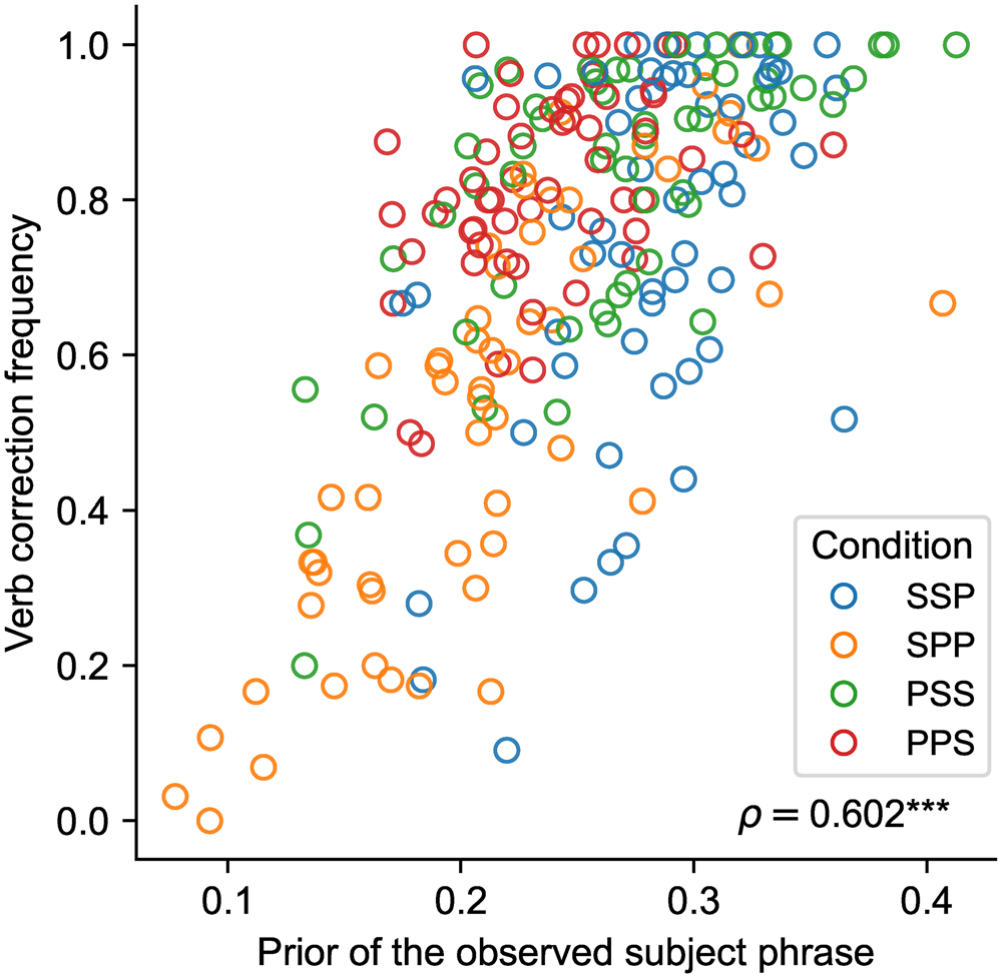
Comparing frequency of verb correction against the estimated prior of the error‐free version with the observed subject phrase. Each hollow circle represents a specific combination of item and mismatch condition. Spearman correlation coefficient ρ between the prior and verb correction frequency is annotated in the panel, with asterisks indicating the significance level (*p<.05, **p<.01, ***p<.001). Note that the noisy‐channel theory does not predict a perfect fit between prior probability and correction frequency, due to the additional role of the error likelihood (Fig. 6).

Additionally, we introduce a temperature parameter α over the prior P(M) in the models that serves as a test of the effect of the prior (see Section 3 for details). A posterior on α close to 0 would suggest that comprehenders are not taking into account variation in the prior. For the full model, the posterior mean of α is 4.146 (95% CI:[3.694, 4.590]), suggesting a strong effect of the prior, consistent with what we see in the simpler visualization of Fig. 5. Note that the inferred temperature parameter α would be around 1 if the prior estimated from the norming task is perfectly calibrated. Our finding of a higher value for the best‐fitting α might indicate that comprehenders overweight the prior relative to what ideal rational behavior would predict, but the finding might alternatively reflect limitations of our method for eliciting prior expectations (Franke et al., 2016; Zhang & Maloney, 2012). Taken together, these results provide quantitative evidence that humans are sensitive to prior probabilities when correcting errors in linguistic input.

#### 4.2.2 Comparing priors normed by people and derived from GPT‐2

In addition to these human rating‐derived priors in the norming study, we also fit models with prior probabilities extracted from a well‐established neural language model. This analysis serves as a way to examine the robustness of the found prior effect to the procedure of estimating the priors. Specifically, we ran the same set of analyses using prior probabilities derived from the GPT‐2 language model (Radford et al., 2019), which has been broadly successful in predicting human behavioral (Oh & Schuler, 2023; Shain, Meister, Pimentel, Cotterell, & Levy, 2024) and neural responses (Caucheteux & King, 2022; Schrimpf et al., 2021) in language comprehension. The GPT‐2 priors were derived by simply normalizing within each item the string probabilities of the error‐free versions as evaluated by the GPT‐2 language model. The prior probabilities derived from GPT‐2 are significantly correlated with the prior expectations estimated in the norming study (Spearman ρ=0.650, p<.001). All the qualitative results obtained using the human‐derived norms were also confirmed using GPT‐2 prior probabilities, with the exception that the confidence interval on the prior temperature parameter α was different: 0.564, 95% CI: [0.483, 0.643] (see Supporting Information).

While we observe a similar pattern in the results from the GPT‐2 language model compared to using the prior estimated from human ratings in the norming task, we acknowledge that future studies could benefit from recruiting a larger sample of participants for estimating prior preferences. We also note that while the analyses with probability judgments derived from GPT‐2 provide converging evidence for the sensitivity to fine‐grained patterns of prior expectation, we do not conclude that GPT‐2 can simply substitute norming data from human participants. Whether contemporary large language models provide robust estimation of relevant language statistics remains an important question for future work.

### 4.3 Calibration of error likelihood

#### 4.3.1 Reanalysis of error distribution in language production

We re‐analyzed data from three language production studies from Ryskin, Bergen, et al. (2021) and Kandel et al. (2022). We chose these datasets as they were known available during the work and contain detailed trial‐level information for estimating the distribution of error types given a specific communicative intent. While prior research (Brehm et al., 2022) aggregated a great amount of datasets from studies of subject–verb agreement production, the error counts reported in previous experiments are still too coarse‐grained for estimating the error likelihood for our purpose. Our reanalysis is based on multi‐level statistical modeling that integrates information from multiple studies. The statistical model assumes that the observed production errors in each experiment arise from a study‐specific multinomial distribution, whose parameters θ (e.g. a probability vector that specifies the rates of different error types) are a noisy version of a cross‐study error likelihood μ, with added Gaussian noise in the log probability space. We implemented the multi‐level model in Stan and estimated the cross‐study error likelihood μ based on the error counts data from Ryskin, Bergen, et al. (2021) and Kandel et al. (2022), using a Markov chain Monte Carlo‐based inference method. We report the posterior of the cross‐study error likelihood in Fig. 6.

**Fig. 6:**
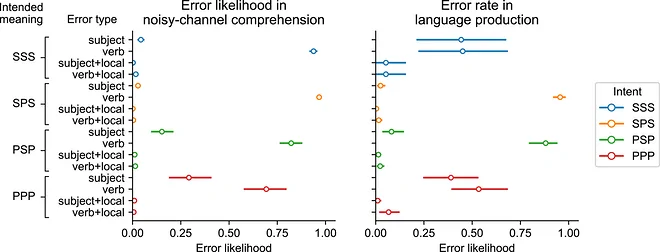
Posterior mean and 95% credible interval of the parameters in the likelihood component estimated from combined data of Study I and Study II, as well as the estimated distribution of errors in language production experiments (Kandel et al., 2022; Ryskin, Bergen, et al., 2021).

#### 4.3.2 Comparing error likelihood to language production statistics

In addition to the effect of prior, we examine how well the representation of error distributions in the human mind is calibrated to the statistics in language production. We plot the estimated noisy‐channel error likelihood parameters of the full Bayesian model in Fig. 6 (left‐hand plot). These parameter estimates can be compared with the actual error distributions in language production, which we estimate using a meta‐analysis of previous language production studies (Kandel et al., 2022; Ryskin, Bergen, et al., 2021) and show in the right‐hand plot of Fig. 6. These paradigms include prefix continuation (Ryskin, Bergen, et al., 2021) similar to the classic study of agreement errors (Bock & Miller, 1991) and scene‐description paradigm with novel objects (Kandel et al., 2022). Crucially, our estimates show a strong overall correspondence between the comprehender's noisy‐channel error likelihoods and the error likelihoods in actual language production, suggesting a strong case against the simplicity‐based theoretical proposal for the error models in the human mind. Across all conditions, verb number errors are overall the most likely type of error. The communicative intent most likely to give rise to subject errors is plural‐subject–noun, plural‐local‐noun (PPP); plural‐subject–noun, singular‐local‐noun (PSP) is second most likely. Errors involving the local noun are rare across the board. These results indicate that comprehenders' models of language production error distributions are generally well calibrated to actual error distribution patterns in the linguistic environment.

There is one case of apparent discrepancy between comprehenders' error likelihoods and actual production error rates, namely when the communicative intent has a singular subject noun and a singular local noun (SSS). However, overall error rates in this situation are by far the lowest for all communicative intent types (see Table 2 for the overall rate of production errors estimated from previous human behavioral studies), which could make the error rates more difficult for comprehenders to calibrate due to minimal direct experience.

**Table 2:** Overall rate of production errors across conditions in human language production studies

Study	SSS	SPS	PSP	PPP
Ryskin et al. (2021) Experiment 1a	0.67%	7.58%	1.51%	2.35%
Kandel et al. (2022) Experiment 1	0.95%	24.56%	22.24%	5.87%
Kandel et al. (2022) Experiment 2	0.22%	15.10%	20.18%	3.73%

## 5 Discussion

In this work, we study whether humans are sensitive to the fine‐grained statistical patterns of errors in language production when deriving non‐veridical interpretation from erroneous linguistic input. We employ a paradigm of free‐form error correction in the domain of subject–verb number agreement mismatch. We hypothesize that humans represent a mental model of language use with statistics calibrated to the linguistic environment and draw inferences about likely errors by integrating both priors over the potentially intended messages as well as structure‐sensitive likelihoods of various types of language production errors. We implement Bayesian models to infer the parameters in the error likelihood and compare them to those independently estimated from language production data. The results suggest that humans track major patterns of structure‐sensitive production errors that they might have possibly experienced in the environment, and use these patterns to reverse‐engineer communicative intent and meaning.

Our work is broadly situated in the noisy‐channel theory of language processing (Levy, 2008b). Prior works have focused on the role of uncertainty over linguistic input in deriving incremental sentence processing difficulty (Bergen, Levy, & Gibson, 2012; Hahn, Futrell, Levy, & Gibson, 2022; Levy, 2008b; Levy et al., 2009; Levy, 2011). Previous research qualitatively compares human behaviors of non‐literal interpretation and the prediction of a noisy‐channel model (Gibson et al., 2013). Here, the task of correcting subject–verb agreement mismatches provides a case where the prior over related forms varies across items. Through a combination of behavioral experiments and Bayesian modeling, our model yields quantitative item‐specific predictions for the type of inferred error and correction that a comprehender may arrive at. Previous results have shown online adaptation to speaker‐specific linguistic environments (Ryskin et al., 2018); our results go beyond these to suggest that comprehenders model patterns of errors depending on specific structural context, closely connected to the idea of structure‐sensitive noise (Poppels & Levy, 2016). In the specific domain of processing subject–verb number mismatches, there have been works comparing models of processing difficulty for sentences with agreement errors (Laurinavichyute & von der Malsburg, 2024; Yadav, Smith, Reich, & Vasishth, 2023). Our studies relate to a recent computational work (Paape, Avetisyan, Lago, & Vasishth, 2021) that compares several algorithmic accounts of the number attraction effect in sentence comprehension as well as the misidentification of the subject's number feature. The noisy‐channel inference framework that we lay out here, however, is at the computational level of analysis (Marr, 1982) and offers a rational account of item‐specific variations in people's non‐veridical interpretations.

Our empirical results may also pose challenges for other leading theories of how the comprehenders may interpret sentences in ways that depart from their literal content (although various mechanisms may be involved across tasks (cf. Paape, 2024), in particular memory retrieval‐based processing theories (Lewis, Vasishth, & Van Dyke, 2006) and “good‐enough” processing theory (Ferreira, 2003; Ferreira & Patson, 2007). In memory‐based theories, linguistic input is incrementally analyzed by cue‐based retrieval of the structural position in the preceding context that best matches the features of the current word, which is then integrated into that position. Reading‐time evidence shows that when a distractor noun and the verb match in number, it can obscure subject–verb agreement errors, evidence that has been argued to support a role for cue‐based retrieval in agreement error processing, potentially including “realignment of the grammatical relations closely related to agreement, like subject‐hood or conceptual number” (Wagers et al., 2009). Our results indicate that such realignment is *not* frequent: participants' corrections predominantly involve changes only to the verb, preserving number on both the subject and local nouns (Fig. 3), and almost never involve making the local noun the subject of the sentence.

The “good‐enough” approach argues that language comprehension does not always involve complete syntactic and semantic analysis of linguistic input, often settling for representations that are merely sufficient for the task that the comprehender needs to perform (Ferreira & Patson, 2007). A key pattern motivating the approach is that comprehenders can perseverate on an incrementally built interpretation even when that interpretation turns out to be inconsistent with subsequent input and have a lingering effect on the final interpretation of the full sentence (Christianson, Hollingworth, Halliwell, & Ferreira, 2001). But, if this were the predominant mechanism behind comprehenders' error corrections when subject–verb agreement errors occur, we would expect the preference for correcting verbs to be uniform in strength across items and structural contexts. Instead, we find that the strength of this preference varies across structural contexts (Figs. 6 and S3 in the Supporting Information) and depends on the relative prior probabilities of error‐free sentences in precisely the manner that the environmentally calibrated noisy‐channel theory predicts. We note that some version of the “good‐enough” approach (Ferreira & Patson, 2007) suggests that comprehenders' semantic representation of an entire sentence could be influenced by factors such as the plausibility of thematic roles. The prior in the noisy‐channel model is consistent with this idea, but formally accounts for the role of plausibility in people's inferences.

Our experimental investigation of error processing in language comprehension involved a novel, relatively explicit error correction task. However, there is good reason to believe that our empirical results and theoretical explanation generalize to the handful of other studies that have investigated interpretation of sentences with subject–verb agreement errors (Brehm et al., 2019, 2021; Patson and Husband, 2016), which used diverse methods to measure the propensity of comprehenders to interpret the subject of a sentence with number mismatch as if it had the number of the verb, corresponding in our studies to the relative probabilities of subject correction versus verb correction. This includes one study using a more implicit measure of interpretation, in which participants made a four‐alternative forced choice about the subject of the heard sentence among images depicting one versus two tokens of the subject noun as well as two distractor images depicting one or two tokens of the local noun (Brehm et al., 2021). In all of these studies, verb correction was more likely than subject correction; furthermore, subject corrections were most common in the singular‐subject, plural‐local‐noun, plural‐verb (SPP) condition, a result that was previously attributed to stronger percolation of plural features in the SPP condition compared to the SSP condition. We find both these patterns in our experimental data (Fig. 3), showing broad consistency with these previous studies. Additionally, the environmentally calibrated noisy‐channel theory predicts both these findings. The overall preference for verb corrections follows from the higher likelihood of verb errors than subject errors (Fig. 6); the greatest propensity follows for subject correction in the SPP condition follows from the combination of two facts: that subject errors are relatively common in this condition (Fig. 6), and that the prior probability of PPP intended meanings tends to be higher than that of SPS meanings (Fig. S8 in the Supporting Information). Discussions in prior works (Brehm et al., 2021; Patson and Husband, 2016) suggest alternative accounts of the increasing rate of misinterpretation in the SPP than SSP condition, which propose that plural features in the local noun and verb may percolate upward and influence the number representation of the complex subject phrase and hence lead to misidentification of the number feature of the head of the subject. However, it remains to be further developed in this account how the specific feature percolation process might generate systematic item‐level variations as shown in Fig. 5.

While we used a novel free‐form error correction task to probe people's noisy‐channel inferences, we recognize the possibility for future studies to employ other more implicit measures. Particularly of interest are eye movement patterns and electrophysiological responses, such as the P600 component of the event‐related potential (ERP). For example, Brehm et al. (2021) combine the visual‐world paradigm and comprehension questions to study how non‐veridical interpretation unfolds in time. Tanner, Nicol, and Brehm (2014) used P600 to examine the nature of number attraction effects in comprehension and production. Prior experimental and modeling works have also suggested P600 as an index of the inferential process of error correction (Li & Ettinger, 2023; Li & Futrell, 2024; Ryskin, Stearns, et al., 2021). Future work could use these behavioral and neural measures to investigate the time course of noisy‐channel inferences as people process the stimuli incrementally. The error correction task we designed in the current work is relatively more explicit but also a less noisy measure compared to eye‐tracking or EEG studies. Since our research questions focus on the fine‐grained inferential patterns, the error correction task provides a straightforward and suitable measure of the content in people's inferences.

We would like to note that here we investigated issues regarding the *environmentally calibrated noisy‐channel processing* hypothesis in a specific domain of correcting subject–verb agreement mismatches. While this domain is useful for putting the theory on a strong test against alternative proposals that parameterize error likelihood based on parsimonious principles, other issues remain open for future research both outside and within the specific domain. First, the experiments focus on a case study of error correction preferences in a specific linguistic construction. Future experimental and computational work is needed to test the generalization of the hypothesis that comprehenders calibrate their mental model of errors to the statistical pattern in the language production environment. Second, we empirically estimated the prior over the communicative intent through an explicit judgment of error‐free forms. While this design helps us circumvent the complex issues between the choice of utterances and the intended meaning, it is an open question whether and how people consider structural preferences in language production during error correction, especially when there are multiple acceptable expressions given a semantic content. For example, imagine a person is trying to communicate a scenario where each bottle has a label versus some of the bottles have a label on them and some do not, would speakers be more likely to utter “The label on the bottles” than “The labels on the bottles” in the former case and show the opposite preferences among these two variations in the latter case? If there is a reliable and salient preference, would people be sensitive to the preference among alternative expressions in error correction? We do not have relevant data that can directly comment on this issue, but it raises several interesting questions for future directions. Third, our hypothesis of the error likelihood assumes that people are sensitive to the potential differences in the error type distribution given specific linguistic structure at the level of abstract number feature, such as a complex subject phrase with a plural head and a plural local noun. It may be possible that people are even sensitive to potentially differential patterns of errors across items. Testing this idea would require measuring noisy‐channel inferences and patterns of production errors for the same set of linguistic materials. Future research could further explore this possibility.

Our work in turn raises the question of how comprehenders' models become calibrated to the statistics of the linguistic environment. Linguistic experience likely plays an important role, both for estimating prior probabilities and the rates of language production errors, but other mechanisms may also be responsible. Common‐sense knowledge may help determine prior probabilities. For language production error likelihoods, comprehenders might internally run forward‐modeling simulations (Pickering & Garrod, 2013) of their own language production mechanisms, with the statistics of those internal simulations calibrating the comprehension noise model. If this latter possibility is true, then the rich debate over the mechanisms underlying agreement errors in language production (Bock, Eberhard, Cutting, Meyer, & Schriefers, 2001; Brehm et al., 2021; Brehm et al., 2022; Patson and Husband, 2016; Ryskin, Bergen, et al., 2021; Schlueter, Parker, & Lau, 2019; G. Smith, Franck, & Tabor, 2018; Wagers et al., 2009) may also advance our understanding of language comprehension. Computational models of agreement production errors (Brehm et al., 2022; G. Smith et al., 2018) could be integrated as a part of an algorithmic account of noisy‐channel language processing. Note that the internal simulations of a forward model may be distinguished from inner speech (Oppenheim, 2013). Open questions remain regarding how a forward model is actually instantiated and whether it is based on the exact same mechanism involved in overt language production (including speech and writing), or instead employs a separate and approximate characterization of overt production. Finally, we note that our theory makes no commitments as to the degree to which noisy‐channel comprehension involves explicit or conscious knowledge or reasoning. These issues remain open for future research.

At a higher level, our work connects to the broader notion of rationality (Anderson, 1991) for understanding human cognition. Going beyond previous evidence regarding parsing and prediction in language processing, our studies suggest that humans achieve robust comprehension of erroneous input by deploying statistical knowledge of errors in language production that is finely calibrated to the linguistic environment. The experimental and computational approach we introduced here also offers a paradigm for quantitatively investigating the relationship among prior experience, mental model of errors, and inference. We believe this approach can be used more broadly to derive quantitative predictions regarding the structure and origin of our mental models of errors in a noisy environment.

## Supporting information



Supporting Information

## Data Availability

Materials, code, and data are available at https://github.com/pqian11/rational‐inference‐from‐agreement‐mismatch.

## Notes

1 We note that the error correction task was different from a traditional rewriting task. Here, in both Study I and II, participants directly interacted with the linguistic stimuli presented on the screen, and the interface traced the changes with respect to the original stimuli. This experimental setup was designed to minimize potential compounding errors from other cognitive processes, such as memory, involved in the execution of the task.

2 The coding pipeline first examined whether the specific singular/plural alternative form of the subject noun, local noun, and the main verb in the stimulus was present in the participant's response, to determine the error correction type. The annotated part‐of‐speech tags were further used to help identify the grammatical number of the main verb and whether the number mismatch was resolved, when the tense and aspect of the main verb were changed in the post‐editing responses, such as changing from the stimulus “The directors of the films has planned …” to “The directors of the films planned …”. The tagger uses the Penn Treebank tag set, which distinguishes various verb forms. The part‐of‐speech tags were involved in the pipeline for 6.67% of the trials in Study I and 3.33% of the trials in Study II. Based on a manual check of the tagging results for a random sample of 50 trial data that were annotated in the pipeline, the tagger achieved an accuracy of 94%. We note that the nltk part‐of‐speech tagger was reasonably good for scaling the coding pipeline, with few distortions introduced into the annotations.

3 We note here that the model only generates agreement mismatch, as E is defined over subject error, verb error, subject+local error, and verb+local error. We explored a variant of the model that adds additional free parameters by allowing E to take the value of “no error.” Our exploratory results suggests that the behavioral data as collected with the specific items through the free‐editing paradigm may not provide sufficient information to estimate the general rate of errors in production.
