# Supplementary material for: Comprehenders' Error Correction Mechanisms Are Finely Calibrated to Language Production Statistics
Source: Cogn Sci. 2026 Oct 30;50(11):e70277. doi: 10.1111/cogs.70277 (PMC13721780; doi:10.1111/cogs.70277)
Supplement: Supplementary file 1 [file COGS-50-e70277-s001.pdf]

Supplementary Information for Comprehenders' Error Correction Mechanisms are Finely Calibrated to Language Production Statistics

A Model Comparison

Fig. S1 plots human correction preference against the correction preferences derived from the fitted models for Study I and II respectively. For both Study I and II, the full model achieves the lowest MSE and highest Spearman correlation with human data, consistent with the results in the modelling analysis on data combined from the two studies.

In addition, we estimated ELPD via k-fold cross-validation for the full model and structure-insensitive model on participant data from Study I and Study II separately. We randomly partitioned all the trial data from a study into eight folds, based on item clusters. Each fold included the trials for a specific subset of 7 different items out of the 56 items. This setup allows us to hold out data at the item level, to measure the model's performance to predict completely new items. The cross-validation estimator of ELPD is defined as:

$$\widehat{\text{ELPD}}_{\text{k-fold}} = \sum_{i=1}^{N} \log \left(\frac{1}{S} P(E_i | O_i, \Theta_{\mathcal{M}}^{(s,-k)}) \right) \tag{S1}$$

where the pointwise log predictive density for the ith trial data (participant's error correction response E_i given the stimulus O_i, in the kth fold) was approximated through averaging over S number of MCMC samples. Each sample $\Theta_{\mathcal{M}}^{(s,-k)}$ was drawn from the posterior $P(\Theta_{\mathcal{M}}|\mathcal{D}^{-k})$ of model $\mathcal{M}$ conditioned on the data $\mathcal{D}^{-k}$ that held out the kth fold where the ith trial data was assigned. We ran sampling-based inference in `stan` [1] with 4 chains and 8000 samples per chain. In total, we collected $S = 32000$ MCMC draws for each of the model parameters, together with random effects $\mathbf{b}_{\text{subject}}$ and $\mathbf{b}_{\text{item}}$. The ELPD difference between the full model and the structure-insensitive model (ΔELPD), as well as the standard error (SE) of the difference, were shown in Table S1 and S2.

B Effect of Prior

We plot the verb correction frequency in human behaviors against the prior judgment of the observed subject phrase in the trial estimated from the norming task. For both Study I and Study II, we observe significant correlation between how often one corrects the verb in

Model	ELPD	ΔELPD	$SE_{\Delta ELPD}$
Full	−1713.40	–	–
Structure-insensitive	−1755.11	−41.71	10.83

Table S1: Comparing Expected Log Predictive Density (ELPD) across models based on cross-validation on Study I data.

Model	ELPD	ΔELPD	$SE_{\Delta ELPD}$
Full	−1780.59	–	–
Structure-insensitive	−1838.87	−58.29	12.06

Table S2: Comparing Expected Log Predictive Density (ELPD) across models based on cross-validation on Study II data.

Study I

Condition	ΔELPD	SE
SSP	−2.76	3.60
SPP	−25.58	7.46
PSS	−1.78	4.21
PPS	−11.58	5.52

Study II

Condition	ΔELPD	SE
SSP	−25.01	6.53
SPP	−18.99	6.30
PSS	−3.78	4.12
PPS	−10.51	6.76

Table S3: Comparing ΔELPD between structure-insensitive model and the full model across trials of different stimulus condition.

the mismatch and how likely the resulted error-free version from verb correction is preferred *a priori*, as shown in Fig. S2. In addition, we examined the posterior of the temperature parameter α over the prior $P(C)$, as introduced in the models to serve as a test of the effect of prior (see *Materials and Methods* for details). For the full model, the posterior mean of α is 3.978 (95% CI:[3.454, 4.490]) for Study I and 4.215 (95% CI: [3.671, 4.755]) for Study II. Consistent with the analysis on data combined from the two studies, these results suggest that human's error correction preferences are strongly sensitive to the prior over error-free forms of the intents.

C Calibration of Error Likelihood

Fig. S3 visualizes inferred error likelihood of the full models fitted on Study I and Study II data respectively, as well as frequency of specific error types in language production studies. Overall, we see a general pattern of a higher verb error rate in the inferred error likelihood parameters, consistent with the error distribution pattern in language production data. Furthermore, we notice higher subject error rate in PPP, which is also reflected in individual language production study.

D Random Split-half Analysis

To evaluate how well the full Bayesian model is, we conducted a random split-half analysis on combined error correction data from Study I and II. We randomly split the combined data into two halves, and compute the Mean Squared Error and Spearman correlation coefficient ρ between error correction frequency estimated from each half. Fig. S4 visualizes the distribution of Mean Squared Error and Spearman correlation coefficient ρ from 3000 simulation of random splits. The MSEs and Spearman ρs of full model fitted on Study I data and Study II data respectively, as annotated in Fig. S1, fall under the likely region of the distribution, suggesting that the goodness of fit of the full Bayesian model is about as good as one would expect given the intrinsic measurement error in probability estimation in the error correction tasks.

E Notes on the Variable Error Rate in the Model

The error type variable E in the full model presented in the paper includes four different types, subject, verb, subject+local, verb+local. Theoretically, once may consider a more complex model where E can also be "no error", in addition to the four error types. In other words, this means that we add a variable ϵ to the model to indicate whether an error occurs or not:

$$P(E, \epsilon, M|O; \Theta, \beta, \alpha) \propto \mathbb{1}(O|M, E, \epsilon)P(E|M, \epsilon; \Theta)P(\epsilon|M; \beta)[P(M)]^\alpha$$

However, while adding "no error" as a possibility to the full model allows the overall error rate to vary across different intents, the model becomes only partially identifiable, since the error correction data may not be able to sufficiently constrain the inference about absolute overall error rate. Fig. S5 shows the posterior of the overall error rate $P(\epsilon|M)$. Fig. S6 shows the normalized error type distribution $P(E|M, \epsilon)$ given an error occurs. The overall pattern of error distribution is similar to that in the full model with constant overall error rate. As there is no strong evidence that the overall error rates vary across communicative intents in the generative model that humans deploy for error correction, we present the results from the models that drop the additional term of "no error", which is equivalent to assuming the same overall error rate across communicative intents.

F Comparing Human-normed to Language Model-based Prior Probabilities

We use the same prior norming materials and compute the probability assigned by GPT-2 [2], a neural language model pre-trained on large amount of text data. For each of the items in our study, we compute the probability of the four possible intents for that item, SSS, SPS, PSP, and PPP, as assigned by the language model. The probabilities for these four intents were then normalized within each item. Fig. S7 visualizes the comparison between model and human-normed prior. Overall, the prior derived from GPT-2 is significantly correlated with human judgment (Spearman $\rho = 0.650$, $p < 0.001$).

We also performed the same set of Bayesian modelling analysis with the priors normed by GPT-2. Fig. S9 shows the comparison between human's error correction preferences and that derived from fitted models. In general, we observe similar pattern in terms of model comparison: the full model provides the best fit of the human data compared to ablated models. In addition, the performance of the full model with GPT-2 normed prior (see Fig. S9) is still worse than the full model with human-normed prior (see Fig. S1), suggesting the potential gap between human prior judgment and the judgment derived from neural language models.

G Materials

G.1 Critical items in error correction tasks

1. The [location/locations] of the [star/stars] [has caught/have caught] attention of some astronomers.
2. The [sound/sounds] of the [string/strings] [is/are] so gorgeous on this guitar.
3. The [cause/causes] of the [problem/problems] [was/were] investigated thoroughly.
4. The [review/reviews] of the [exhibition/exhibitions] [is/are] too pedantic for the readers.
5. The [speech/speeches] of the [candidate/candidates] [is/are] summarized in the news show today.
6. The [test/tests] of the [device/devices] [was/were] carried out before packaging.
7. The [director/directors] of the [film/films] [has planned/have planned] to hold an audition this week.
8. The [teacher/teachers] of the [student/students] [is/are] attending a workshop on active learning.
9. The [photo/photos] of the [mountain/mountains] [is/are] displayed in an exhibition at the gallery.
10. The [editor/editors] of the [book/books] [has been/have been] invited to the seminar.
11. The [host/hosts] of the [show/shows] [was/were] nominated for an award.
12. The [judge/judges] of the [competition/competitions] [is/are] the same as last year.
13. The [friend/friends] of the [actor/actors] [has/have] come to see the play.
14. The [bone/bones] of the [dinosaur/dinosaurs] [is/are] on display at the Natural History Museum.
15. The [doubt/doubts] about the [product/products] [was/were] completely expected.

16. The [article/articles] about the [discovery/discoveries] [is/are] very easy to read.
17. The [story/stories] about the [scientist/scientists] [has inspired/have inspired] many people.
18. The [alarm/alarms] in the [station/stations] [has been/have been] maintained regularly.
19. The [musician/musicians] in the [orchestra/orchestras] [is/are] organizing a community concert.
20. The [character/characters] in the [episode/episodes] [was/were] too chaotic.
21. The [picture/pictures] in the [album/albums] [reminds/remind] them of the days they spent together.
22. The [player/players] in the [game/games] [has/have] won several championships.
23. The [bee/bees] in the [flower/flowers] [is/are] collecting pollen.
24. The [rug/rugs] under the [chair/chairs] [is/are] handmade from wool and silk.
25. The [bench/benches] under the [tree/trees] [is/are] cleaned everyday.
26. The [tunnel/tunnels] under the [lake/lakes] [has cost/have cost] a lot of money beyond enigeering challenges.
27. The [cafe/cafes] near the [shop/shops] [has got/have got] popular around the neighbourhood.
28. The [key/keys] to the [office/offices] [is/are] on the table.
29. The [door/doors] to the [room/rooms] [looks/look] very festive with the decorations.
30. The [tour/tours] to the [island/islands] [was/were] suspended temporarily.
31. The [visit/visits] to the [garden/gardens] [has/have] sparked her interest in botany.
32. The [letter/letters] to the [editor/editors] [is/are] published online.
33. The [applicant/applicants] to the [school/schools] [has received/have received] emails about financial assistance.
34. The [railway/railways] to the [village/villages] [has promoted/have promoted] economic growth.
35. The [trail/trails] to the [pond/ponds] [is/are] highly recommended for beginner cyclists.
36. The [gift/gifts] for the [kid/kids] [is/are] hidden under the bed.
37. The [cake/cakes] for the [family/families] [was/were] prepared by a local bakery.
38. The [ticket/tickets] for the [game/games] [was/were] ordered online.

39. The [exam/exams] for the [student/students] [was/were] challenging but interesting.
40. The [prize/prizes] for the [winner/winners] [is/are] a big surprise.
41. The [plan/plans] for the [event/events] [was/were] discussed at a group meeting.
42. The [wine/wines] on the [table/tables] [is/are] from Napa Valley.
43. The [magazine/magazines] on the [shelf/shelves] [was/were] moved to a different place.
44. The [pebble/pebbles] on the [beach/beaches] [is/are] up to 200 million years old.
45. The [label/labels] on the [box/boxes] [is/are] positioned properly before shipping.
46. The [painting/paintings] on the [wall/walls] [was/were] taken down during the renovation.
47. The [slogan/slogans] on the [poster/posters] [is/are] printed in larger font.
48. The [debate/debates] with the [lawyer/lawyers] [is/are] broadcast live on the radio.
49. The [contract/contracts] with the [company/companies] [has been/have been] examined carefully by a legal consultant.
50. The [project/projects] with the [team/teams] [has been/have been] very successful.
51. The [rehearsal/rehearsals] with the [band/bands] [is/are] rescheduled.
52. The [lawsuit/lawsuits] against the [company/companies] [was/were] resolved in court.
53. The [vote/votes] against the [measure/measures] [is/are] indicative of the ongoing miscommunication.
54. The [complaint/complaints] from the [customer/customers] [was/were] filed in time.
55. The [question/questions] from the [interviewer/interviewers] [is/are] too difficult.
56. The [bike/bikes] at the [station/stations] [is/are] broken.

G.2 Filler items in error correction tasks

1. The company is looking for an professional architect to design the new building.
2. The researcher came up with an creative approach to solving the problem.
3. It would be an great opportunity for the young artists to showcase their works.
4. The athelete got a very good advice from the coach.
5. She played a piano at the party yesterday.
6. He used to went to school by the bike everyday.

7. The musician appeared nervously when she walked to the stage.
8. The team know that they have to work hardly to lauch the new product in time.
9. The accident was taken place at the junction of two roads.
10. The teacher write a long letter the president.
11. The charity received a large donation the company.
12. She baked a birthday cake her daughter.
13. The family spent all their savings buy the apartment in New York City.
14. He found it really difficult learn a new languages.
15. They finally have decided to with the old plan.
16. The students played yesterday football.
17. The museum houses a collection of furniture and and objects designed by the architect Antoni Gaudi.
18. He really enjoyed the trip with the his old friends to the mountain.
19. This country we have visited many cities.
20. Carry a pile of boxes, his foot caught on a step.
21. Living here for more than ten years, many things have changed.
22. California is home to many national parks, locating in the western of the America and closed to the Pacific ocean.
23. The book was just published won a prestigious award.
24. Take notes when one reads a book would be helpful.
25. There is another article is worth taking a look.
26. How do babies acquire their native languages is still unclear to researchers.
27. The task seems to be overwelming for beginners.
28. The students often staying up late before the final exams.
29. The foods has cooked.
30. Please close the light before leaving the room.

G.3 Items in prior norming task

Listed below are the corresponding items used in the norming study for prior elicitation. Notice that the sentences stay the same as their counterpart among the critical items in the editing task except that the verbs are changed to a different tense or modal context that does not require number inflection.

1. The [location/locations] of the [star/stars] caught attention of some astronomers.
2. The [sound/sounds] of the [string/strings] will be so gorgeous on this guitar.
3. The [cause/causes] of the [problem/problems] should be investigated thoroughly.
4. The [review/reviews] of the [exhibition/exhibitions] might be too pedantic for the readers.
5. The [speech/speeches] of the [candidate/candidates] will be summarized in the news show today.
6. The [test/tests] of the [device/devices] should be carried out before packaging.
7. The [director/directors] of the [film/films] might plan to hold an audition this week.
8. The [teacher/teachers] of the [student/students] will be attending a workshop on active learning.
9. The [photo/photos] of the [mountain/mountains] will be displayed in an exhibition at the gallery.
10. The [editor/editors] of the [book/books] should be invited to the seminar.
11. The [host/hosts] of the [show/shows] will be nominated for an award.
12. The [judge/judges] of the [competition/competitions] will be the same as last year.
13. The [friend/friends] of the [actor/actors] will come to see the play.
14. The [bone/bones] of the [dinosaur/dinosaurs] will be on display at the Natural History Museum.
15. The [doubt/doubts] about the [product/products] would be completely expected.
16. The [article/articles] about the [discovery/discoveries] should be very easy to read.
17. The [story/stories] about the [scientist/scientists] will inspire many people.
18. The [alarm/alarms] in the [station/stations] should be maintained regularly.
19. The [musician/musicians] in the [orchestra/orchestras] will be organizing a community concert.
20. The [character/characters] in the [episode/episodes] might be too chaotic.

21. The [picture/pictures] in the [album/albums] will remind them of the days they spent together.
22. The [player/players] in the [game/games] might have won several championships.
23. The [bee/bees] in the [flower/flowers] may be collecting pollen.
24. The [rug/rugs] under the [chair/chairs] might be handmade from wool and silk.
25. The [bench/benches] under the [tree/trees] should be cleaned everyday.
26. The [tunnel/tunnels] under the [lake/lakes] will cost a lot of money beyond enigeering challenges.
27. The [cafe/cafes] near the [shop/shops] got popular around the neighbourhood.
28. The [key/keys] to the [office/offices] should be on the table.
29. The [door/doors] to the [room/rooms] will look very festive with the decorations.
30. The [tour/tours] to the [island/islands] will be suspended temporarily.
31. The [visit/visits] to the [garden/gardens] might have sparked her interest in botany.
32. The [letter/letters] to the [editor/editors] might be published online.
33. The [applicant/applicants] to the [school/schools] will receive emails about financial assistance.
34. The [railway/railways] to the [village/villages] will promote economic growth.
35. The [trail/trails] to the [pond/ponds] should be highly recommended for beginner cyclists.
36. The [gift/gifts] for the [kid/kids] will be hidden under the bed.
37. The [cake/cakes] for the [family/families] will be prepared by a local bakery.
38. The [ticket/tickets] for the [game/games] must be ordered online.
39. The [exam/exams] for the [student/students] should be challenging but interesting.
40. The [prize/prizes] for the [winner/winners] will be a big surprise.
41. The [plan/plans] for the [event/events] will be discussed at a group meeting.
42. The [wine/wines] on the [table/tables] might be from Napa Valley.
43. The [magazine/magazines] on the [shelf/shelves] will be moved to a different place.
44. The [pebble/pebbles] on the [beach/beaches] could be up to 200 million years old.
45. The [label/labels] on the [box/boxes] should be positioned properly before shipping.

46. The [painting/paintings] on the [wall/walls] will be taken down during the renovation.
47. The [slogan/slogans] on the [poster/posters] should be printed in larger font.
48. The [debate/debates] with the [lawyer/lawyers] will be broadcast live on the radio.
49. The [contract/contracts] with the [company/companies] must be examined carefully by a legal consultant.
50. The [project/projects] with the [team/teams] will be very successful.
51. The [rehearsal/rehearsals] with the [band/bands] will be rescheduled.
52. The [lawsuit/lawsuits] against the [company/companies] will be resolved in court.
53. The [vote/votes] against the [measure/measures] could be indicative of the ongoing miscommunication.
54. The [complaint/complaints] from the [customer/customers] must be filed in time.
55. The [question/questions] from the [interviewer/interviewers] might be too difficult.
56. The [bike/bikes] at the [station/stations] might be broken.

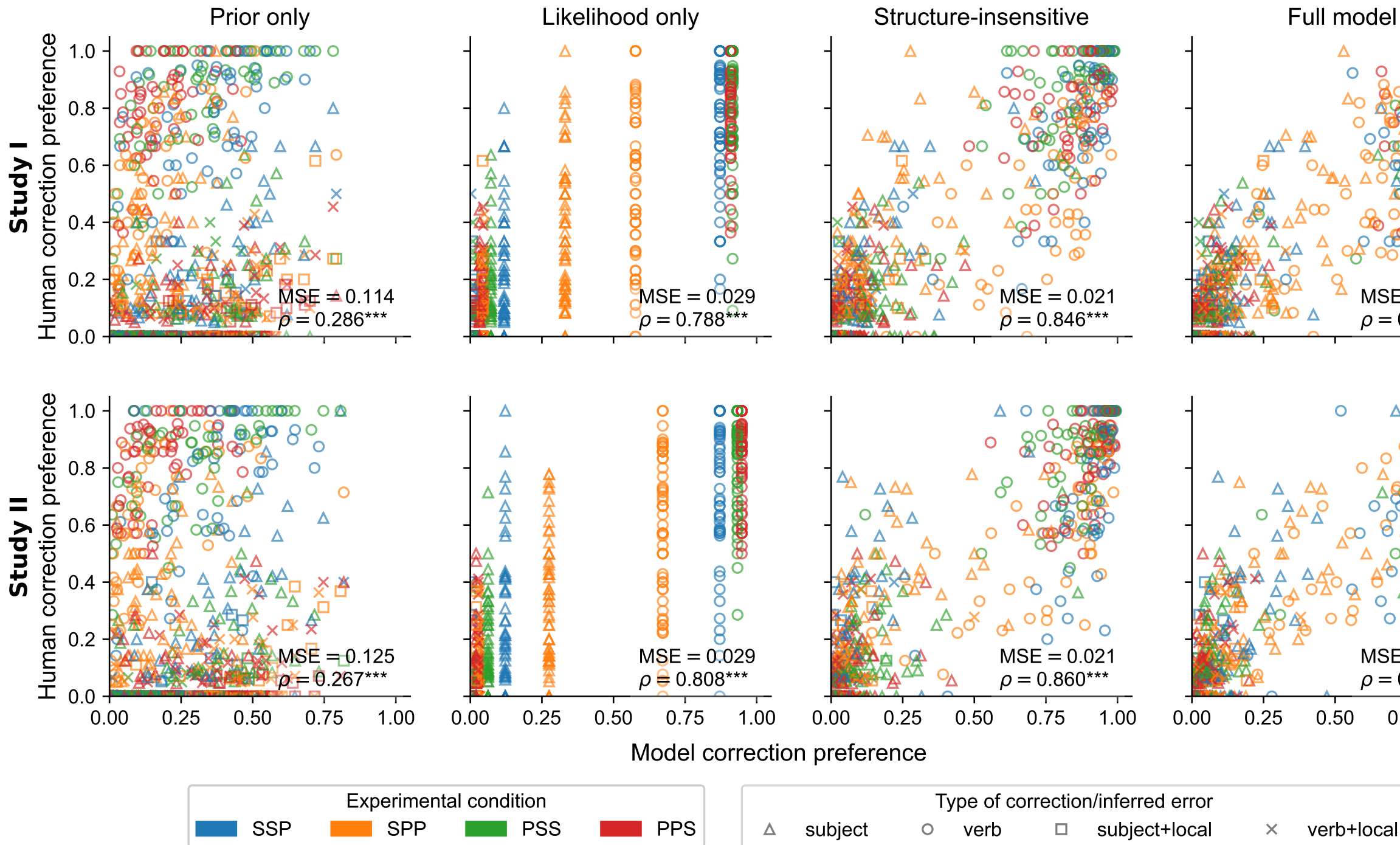

Figure S1: Compare correction preferences derived from fitted models to human correction preferences. Models are fitted on data from the two studies respectively.

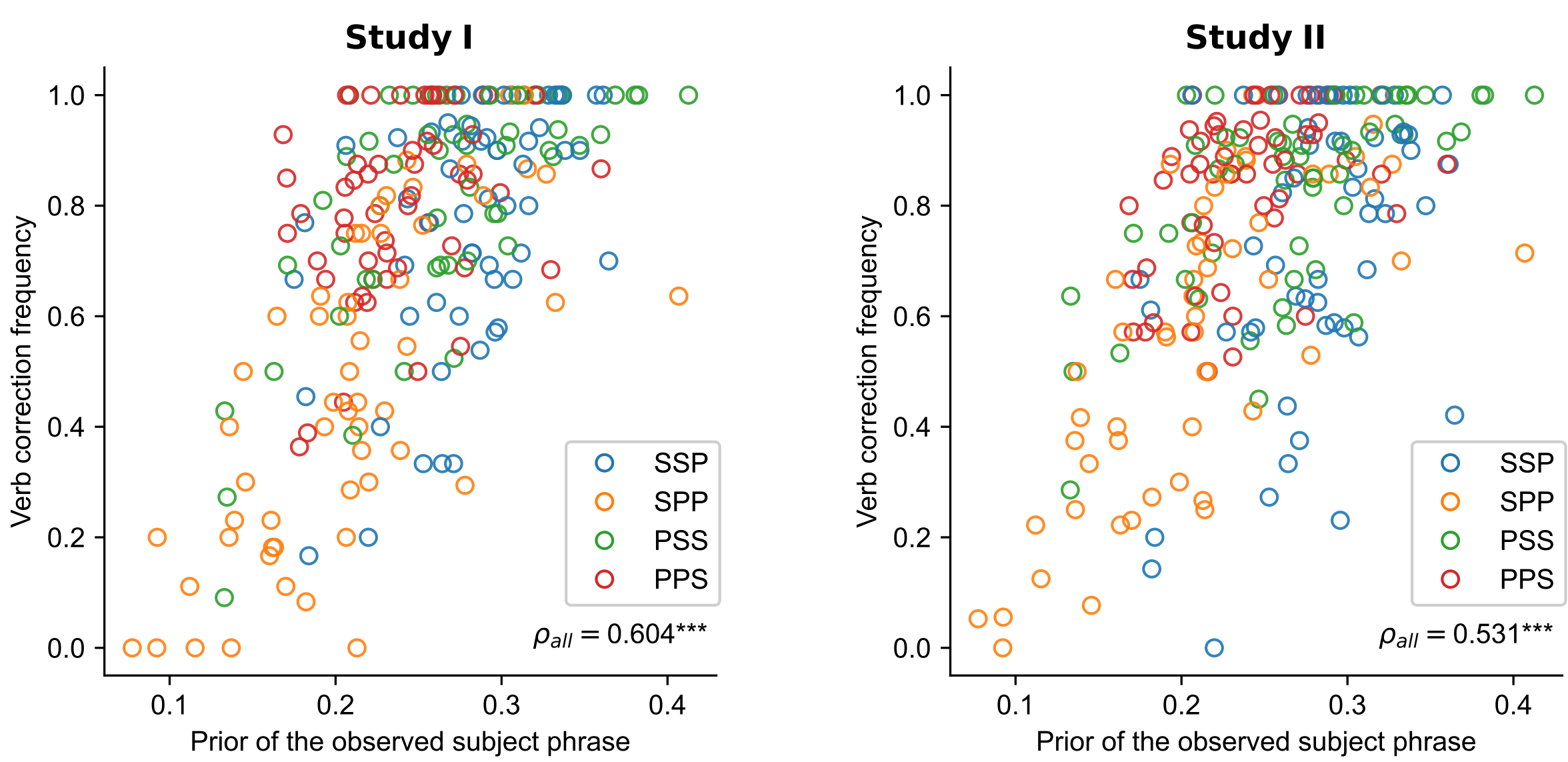

Figure S2: Comparing frequency of verb correction against the estimated prior of the error-free version with the observed subject phrase. Each hollow circle represents a specific combination of item and mismatch condition. Spearman correlation coefficient ρ between the prior and verb correction frequency is annotated in each panel, with asterisks indicating the significance level (*: $p < 0.05$, **: $p < 0.01$, ***: $p < 0.001$).

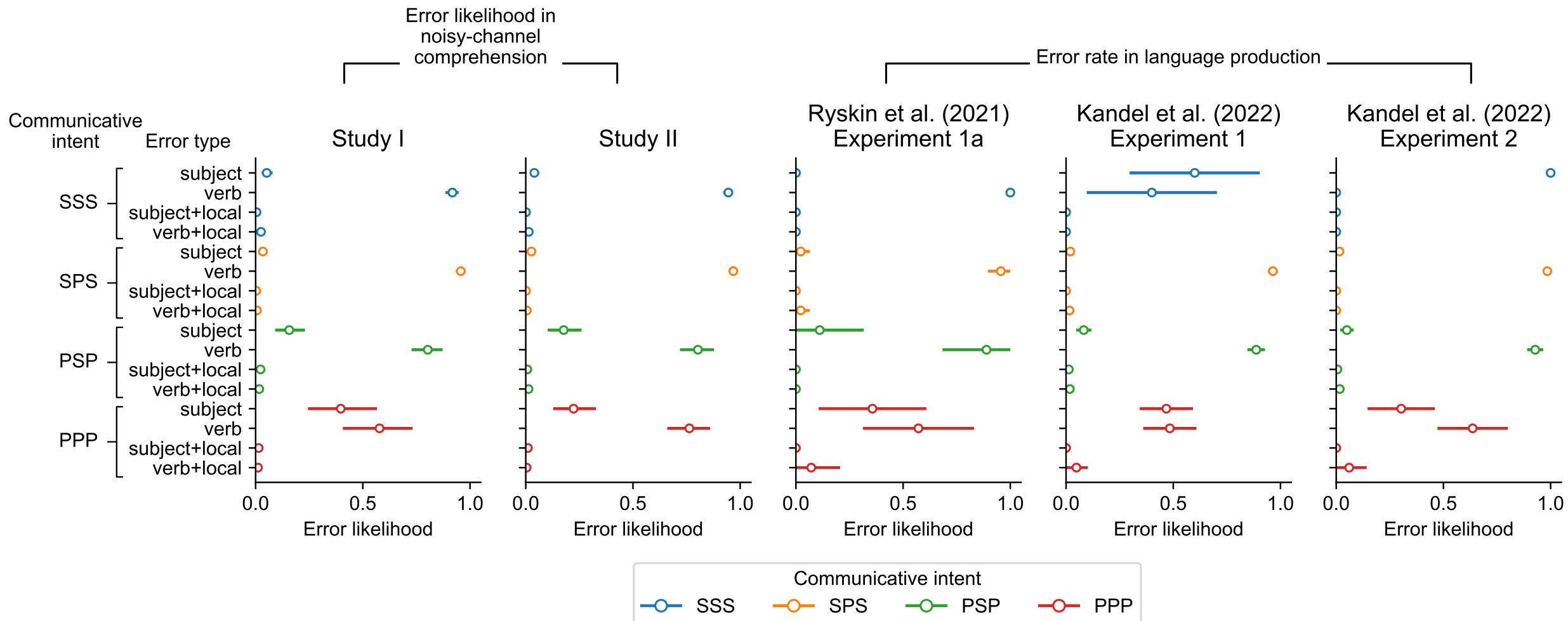

Figure S3: Compare error likelihood inferred from error correction tasks and language production tasks.

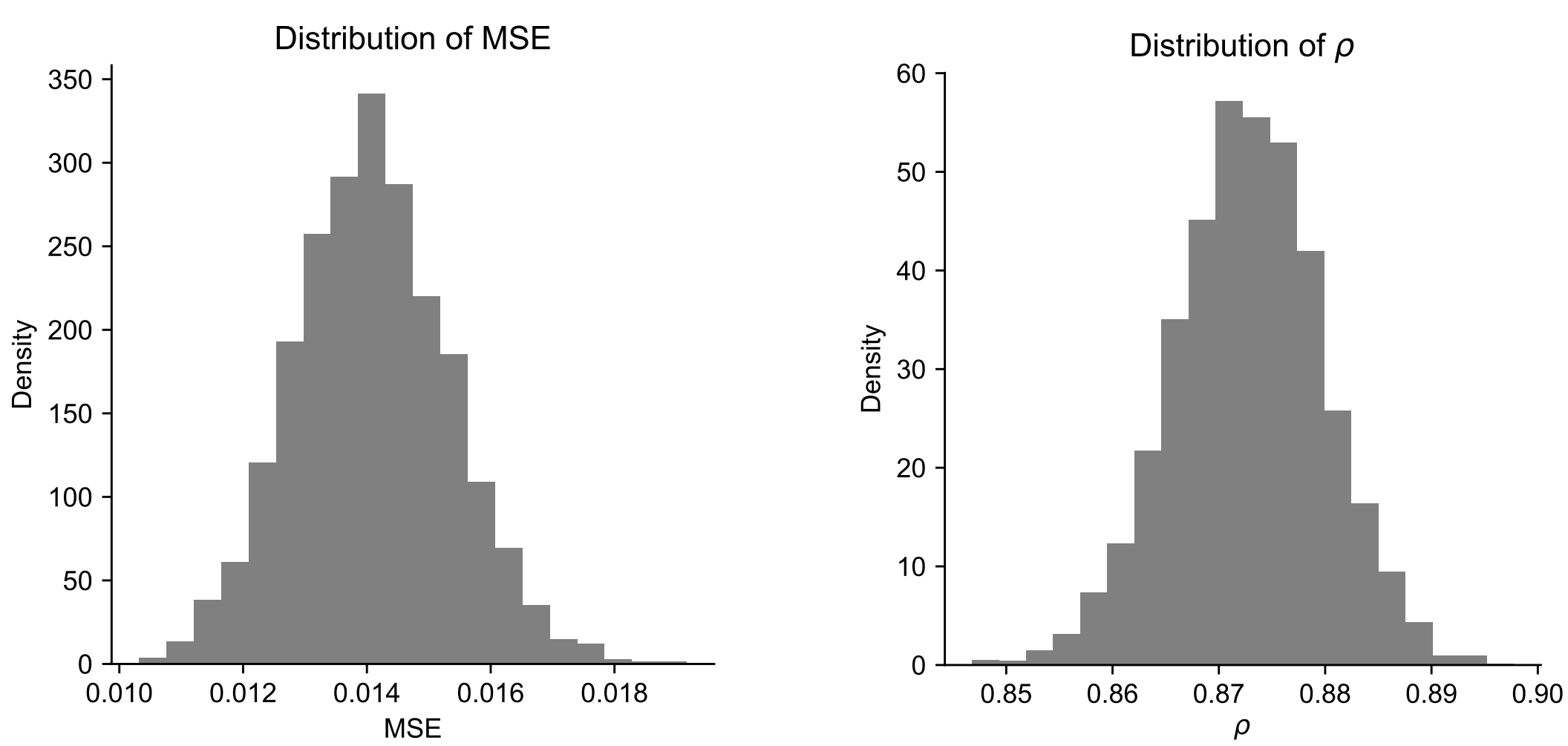

Figure S4: Distribution of Mean Squared Error and Spearman correction coefficient ρ from random split-half analysis of combined data from Study I & II.

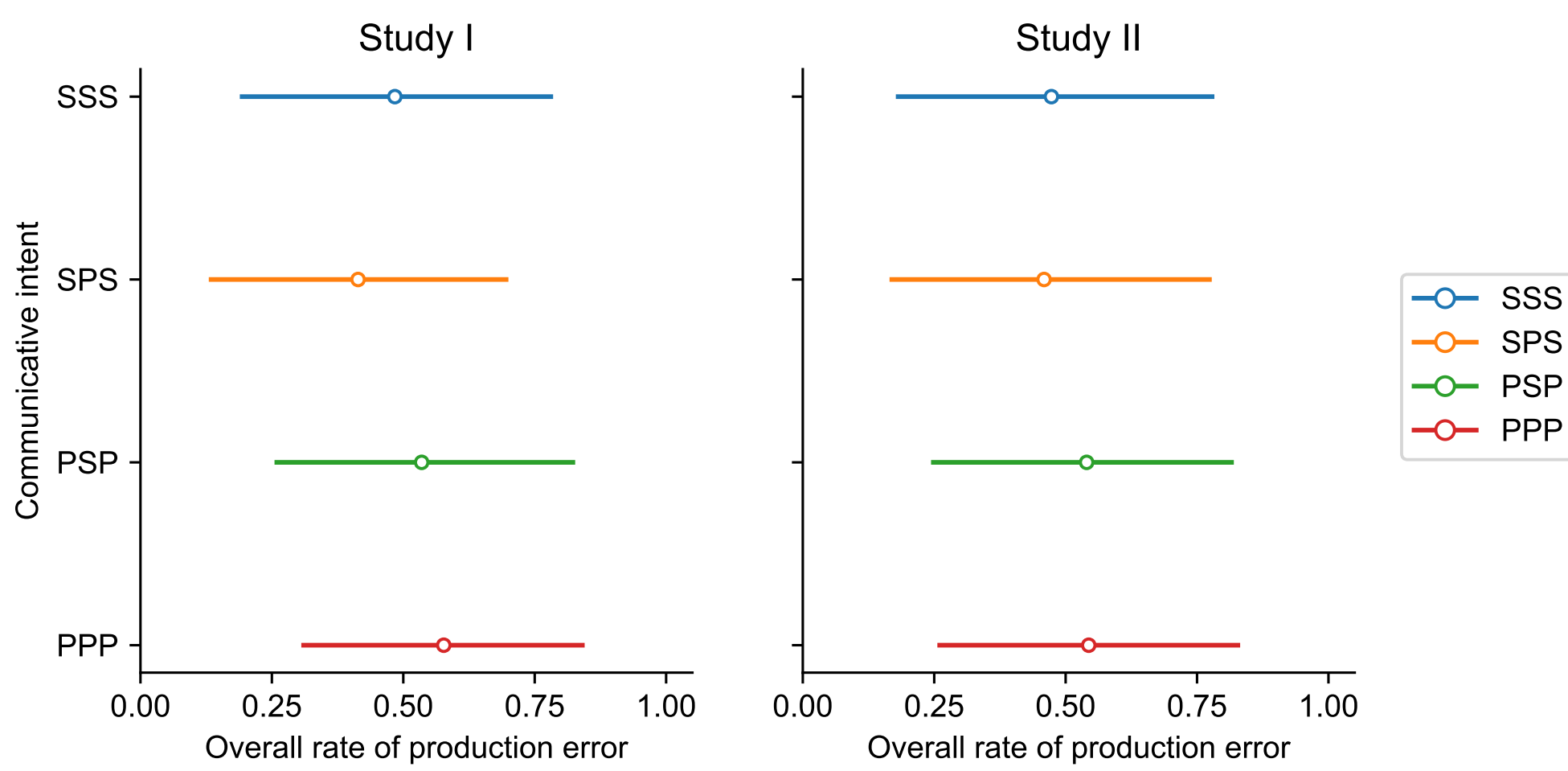

Figure S5: Inferred overall rate of production errors across conditions based on human data in error correction studies.

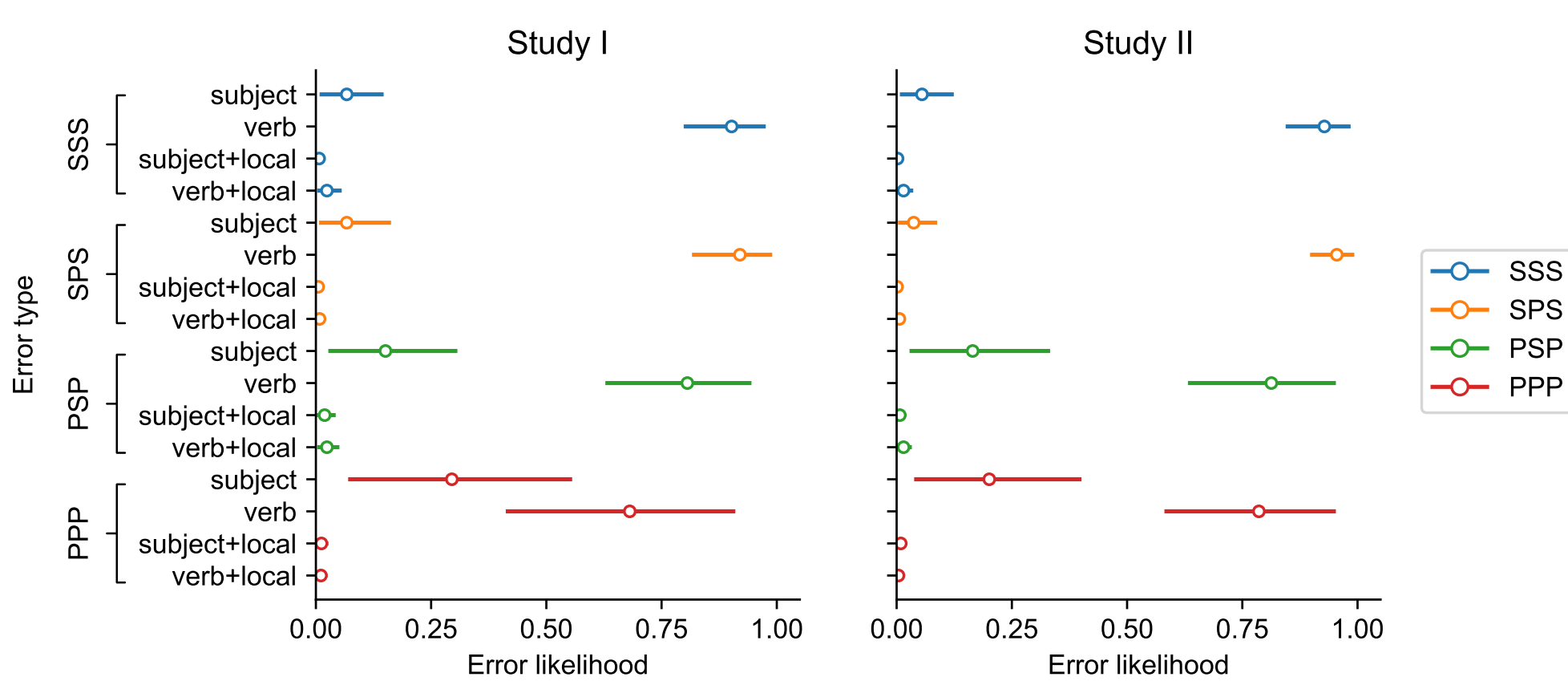

Figure S6: Inferred error likelihood parameter for full Bayesian model extended with unknown rate of production errors.

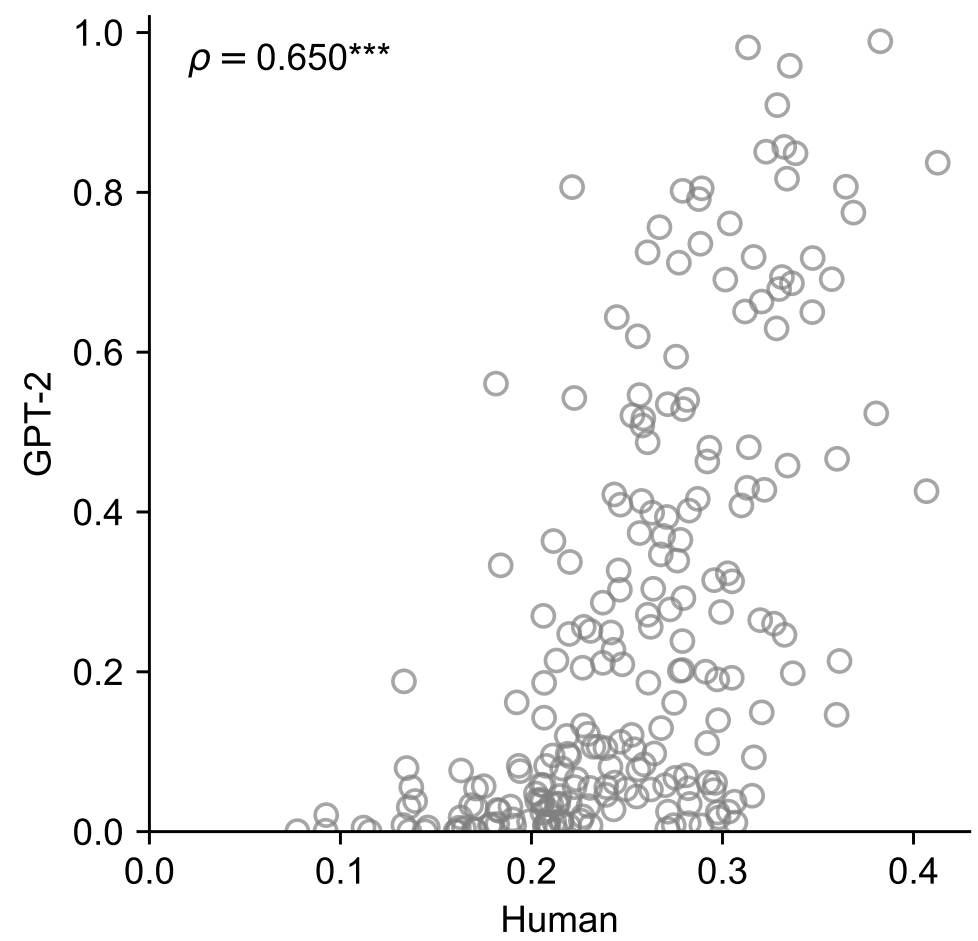

Figure S7: Compare prior probabilities derived from GPT-2, a pretrained neural language model [2], with the prior derived from human norming task. Spearman correlation coefficient ρ between GPT-2 output and human judgment is annotated in the plot.

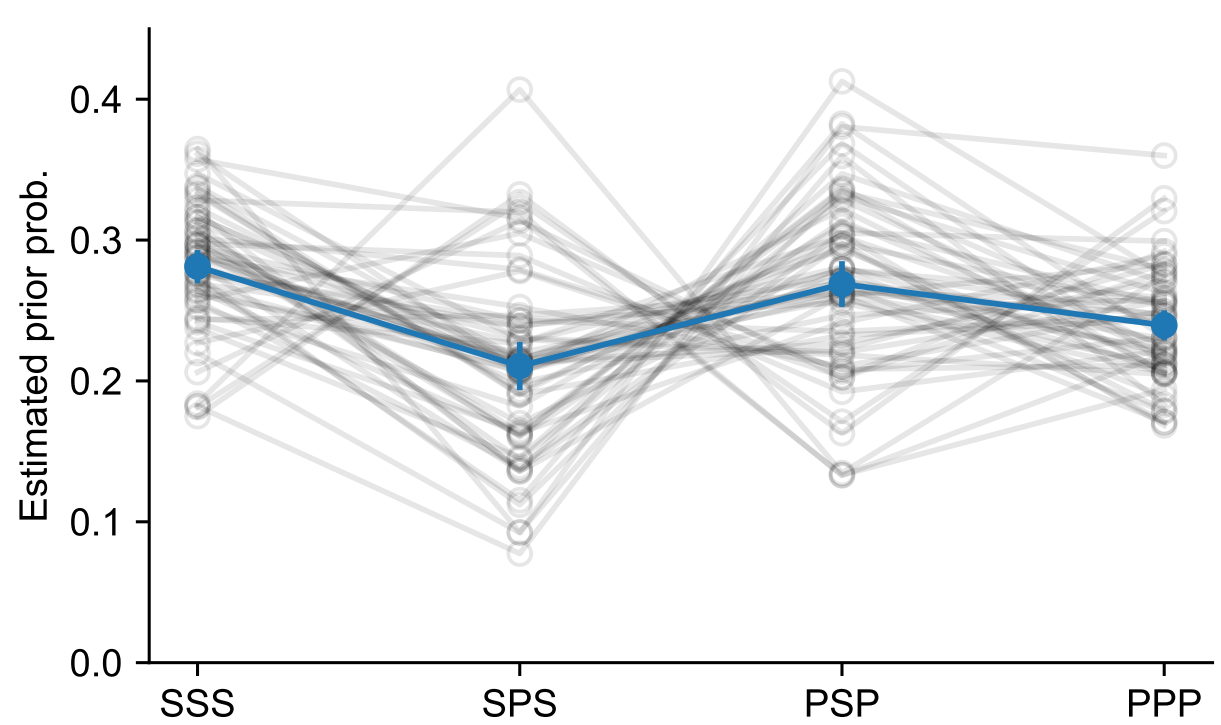

Figure S8: Visualization of human-normed prior probabilities. Gray lines represent item-specific prior probability estimated from participants' judgments in the norming task. Blue solid line represent the overall prior probability average across all 56 items.

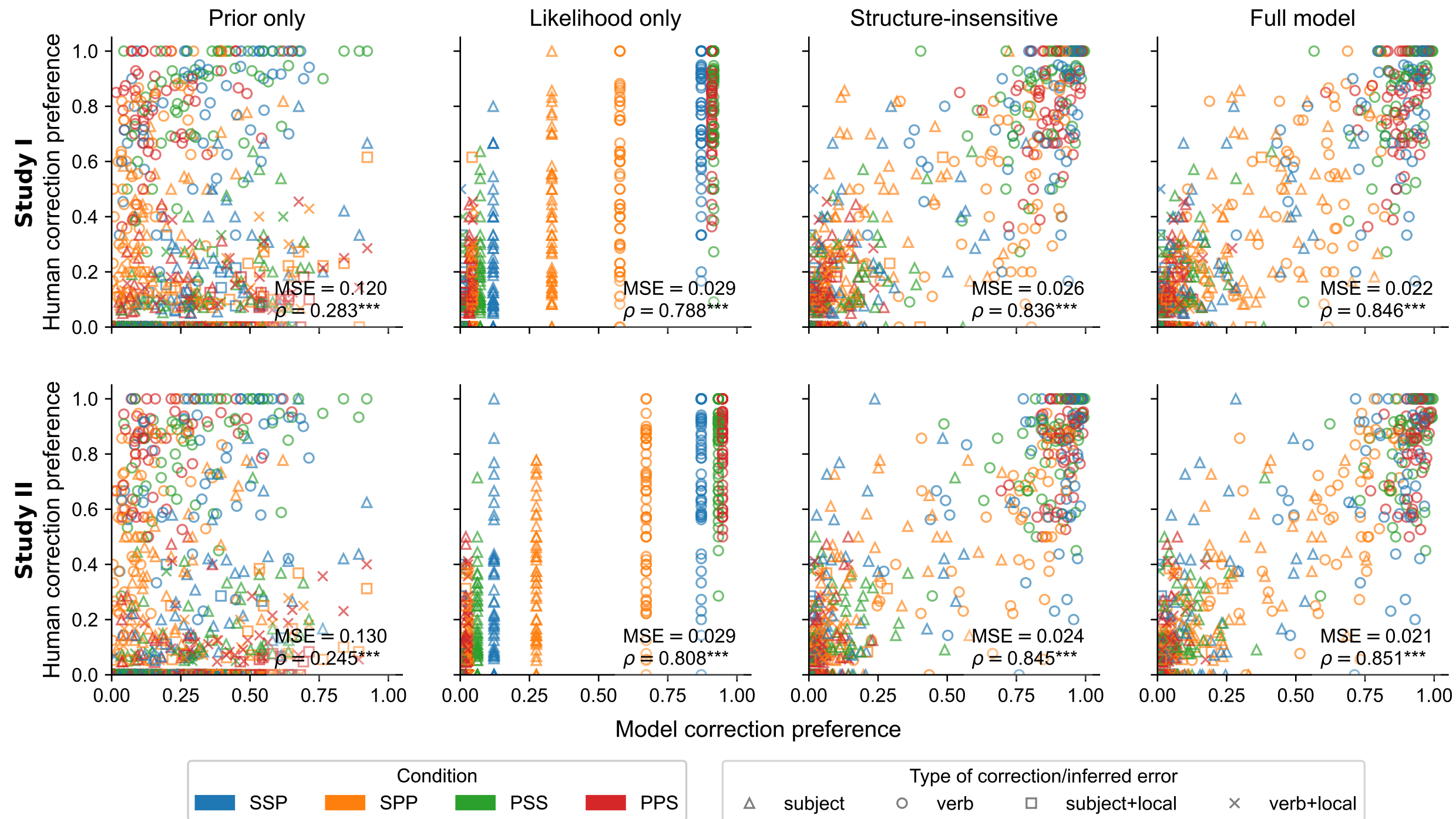

Figure S9: Compare correction preferences derived from fitted models to human correction preferences. Models are fitted on data from the two studies respectively with by-item normalized priors derived from GPT-2.

M	E	$P(O\|M,E)$
SSS	subject	$\mathbb{1}(O = \text{PSS})$
	verb	$\mathbb{1}(O = \text{SSP})$
	subject+local	$\mathbb{1}(O = \text{PPS})$
	verb+local	$\mathbb{1}(O = \text{SPP})$
SPS	subject	$\mathbb{1}(O = \text{PPS})$
	verb	$\mathbb{1}(O = \text{SPP})$
	subject+local	$\mathbb{1}(O = \text{PSS})$
	verb+local	$\mathbb{1}(O = \text{SSP})$
PSP	subject	$\mathbb{1}(O = \text{SSP})$
	verb	$\mathbb{1}(O = \text{PSS})$
	subject+local	$\mathbb{1}(O = \text{SPP})$
	verb+local	$\mathbb{1}(O = \text{PPS})$
PPP	subject	$\mathbb{1}(O = \text{SPP})$
	verb	$\mathbb{1}(O = \text{PPS})$
	subject+local	$\mathbb{1}(O = \text{SSP})$
	verb+local	$\mathbb{1}(O = \text{PSS})$

Table S4: Detailed illustration of $\mathbb{1}(O|M,E)$ as an indicator function given specific communicative intent M and error type E.

References

[1] Bob Carpenter et al. "Stan: A probabilistic programming language". In: *Journal of statistical software* 76.1 (2017), pp. 1–32.

[2] Alec Radford et al. "Language models are unsupervised multitask learners". In: *OpenAI blog* 1.8 (2019), p. 9.
